# Multi-criteria decision support for planning and evaluation of performance of viral marketing campaigns in social networks

**DOI:** 10.1371/journal.pone.0209372

**Published:** 2018-12-27

**Authors:** Artur Karczmarczyk, Jarosław Jankowski, Jarosław Wątróbski

**Affiliations:** 1 Faculty of Computer Science and Information Technology, West Pomeranian University of Technology in Szczecin, ul. Żołnierska 49, 71-210 Szczecin, Poland; 2 Faculty of Economics and Management, University of Szczecin, Mickiewicza 64, 71-101, Szczecin, Poland; Universidad Nacional de Mar del Plata, ARGENTINA

## Abstract

The current marketing landscape, apart from conventional approaches, consists of campaigns designed especially for launching information diffusion processes within online networks. Associated research is focused on information propagation models, campaign initialization strategies and factors affecting campaign dynamics. In terms of algorithms and performance evaluation, the final coverage represented by the fraction of activated nodes within a target network is usually used. It is not necessarily consistent with the real marketing campaigns using various characteristics and parameters related to coverage, costs, behavioral patterns and time factors for overall evaluation. This paper presents assumptions for a decision support system for multi-criteria campaign planning and evaluation with inputs from agent-based simulations. The results, which are delivered from a simulation model based on synthetic networks in a form of decision scenarios, are verified within a real network. Last, but not least, the study proposes a multi-objective campaign evaluation framework with several campaign evaluation metrics integrated. The results showed that the recommendations generated with the use of synthetic networks applied to real networks delivered results according to the decision makers’ expectation in terms of the used evaluation criteria. Apart from practical applications, the proposed multi-objective approach creates new evaluation possibilities for theoretical studies focused on information spreading processes within complex networks.

## 1 Introduction

The evolution of social networking platforms has led to a crucial need to understand how millions of online users behave, including their online and real life behaviors, patterns and predispositions [1]. Apart from studying social relations and online activity, information spreading processes are among the phenomenas with high attention from both researchers and practitioners. In a number of cases, as a result of information spreading, viral marketing seems to produce better results than traditional advertising campaigns [2]. There is an increase in the number of online marketers using this opportunity to place even greater efforts in the engagement of potential consumers to benefit from their services and products by propagating information. Due to an increased trustworthiness of communications within a social network that has ties that are particularly strong, recommendations that are socially oriented have a greater impact on the targeted consumers than traditional commercial messages [3]. The research that is related to diffusion of marketing content takes into consideration the factors that lead to campaigns that are successful [4] [5], factors affecting usersÔÇÖ participation during information spreading [6], the initial seed sets that are selected for the initialization of the campaign [7] [8], as well as using epidemic models to analyze diffusion processes [9]. Other studies emphasize the role of different centrality measures used for the selection of initial influencers [10], the impact of homophily for successful selection of the initial network nodes [11], the role of the content and network structures [12], user motivation to forward content [5], the role of emotions [13] [14] and other factors [15]. Apart from static networks and single layer structures, multilayer networks [16] and the spreading of information in temporal networks have been studied in the more recent research [17].

Many earlier studies were focused on theoretical and empirical approaches increasing the number of reached customers within a network [7] [18]. While it is an important metric of the campaign success, several other factors should also be taken into account [19]. Apart from coverage, they include the campaign’s costs and duration, number of initial seeds and their selection strategies [20] [21].

This study proposes and examines the framework for a multi-objective evaluation of information spreading processes. The presented framework can be used for strategic planning of information spreading processes in order to help selecting the appropriate strategy for selection of the initial nodes within the network and adjusting the number of activated nodes in the seeding process. While viral marketing processes can be based on increasing the motivation of content forwarding, the evaluation of the potential of available approaches creates another areas of applications of the multi-objective methods. The main contribution of the presented study is the framework for multi-objective selection of methods influencing campaign dynamics and coverage with the use of several evaluation criteria. In practical terms, an evaluation model was created with the use of the PROMETHEE II method and agent-based simulations were performed with sensitivity analysis used.

The remainder of this paper provides the methodological background and the conceptual framework in Section 2. This is followed by the example planning process in Section 3, data evaluation and searching for alternatives in Section 4 with conclusions in Section 5.

## 2 Materials and methods

### 2.1 Information spreading in complex networks

Social network marketing strategies are geared to motivating users to pass the advertised product information to their friends and contacts within their social networks. With its interdisciplinary approach, the research that has been done in this field attracts sociologists, physicists, computer scientists and marketers with a wide range of approaches and research goals [7] [9] [3]. The prior research in this field implemented a macroscopic approaches to analyze the quantity of customers acquired using the diffusion of innovations’ mechanics [22] [23]. The processes at the level of social networks, as well as their participants, are monitored at a detailed level, offering a microscopic view. The identification and assessment of those who send and receive messages make the detailed monitoring of the processes involved in the distribution of information possible [24].

The methodological background on network structures evolved simultaneously, yet separately, on various disciplines [25]. In this paper, network G is defined as a set of nodes (vertices) *V*(*G*) interconnected with the set of edges *E*(*G*), which can be represented with the following mathematical notation: *G*(*V*, *E*). A path in graph G is a set of edges {{*v*_1_, *v*_2_}, {*v*_2_, *v*_3_}, …, {*v*_*n*−1_, *v*_*n*_}}, where the end of the {*v*_*i*_, *v*_*i*+1_} edge is the beginning of the {*v*_*i*+1_, *v*_*i*+2_} edge for every *i* = 0, …, *n* − 2, and where every node and edge are unique. The length of a path is the number of edges it comprises of. The distance *d*(*i*, *j*) from node *i* to node *j* is the length of the shortest path from *i* to *j*.

The current research done in the field can be identified as taking different directions. The dedicated solutions like linear threshold [22] and independent cascades model [26], as well as epidemic research models are implemented to model the way information is spread [9]. A large number of studies relate to the initiation of the information distribution processes and network node selection [7]. The most of the seeding strategies use network centrality measures for obtaining the nodes’ ranking and initiating the seeding process, assuming an increased potential to distribute information resulting from the top nodes, having a vital role in the network structures. One of the most fundamental characteristics of a graph’s node is its degree, i.e. the number of edges incident to this node, denoted *deg*(*v*), where *v* ∈ *V*. In case of information spreading, the higher the node’s degree, the more nodes the information can be potentially propagated to. The degree distribution *P*(*k*) of a network represents the fraction of the nodes in the network which have degree equal to *k*. Other measures based on closeness, betweenness or eigenvector centralities are used as well. The closeness of a node is a measure of its centrality in a network, calculated as the sum of the lengths of the shortest paths between the node and all other nodes in the network:

$$\begin{array}{c} C\left(i\right)=\frac{1}{\sum_{j}\;d\left(j,i\right)} \end{array}$$ (1)

The smaller the closeness value, the more central position in the network the node has, thus allowing to reach every other node in fewer steps. For every pair of vertices (*v*_*i*_, *v*_*j*_) in graph G, there exists at least one shortest path between *v*_*i*_ and *v*_*j*_ with the number of edges on the path minimized. The betweenness of a vertex *v*_*k*_ is the ratio of the number of such shortest paths that pass through *v*_*k*_ to all such shortest paths. The higher the betweenness value of a node, the more nodes can be accessed through that node.

Eventually, the eigenvector centrality is a measure of influence of a node in a network. Each node in a network obtains a relative score based on a concept that connections from the high-scored nodes contribute to the node’s score more than connections from the low-scoring ones. Therefore, a high eigenvector centrality value means that a node has more influence on the other nodes in the network.

These approaches tend to be used despite the fact that they require computational resources that are limited and that they fail in the delivery of an optimal seed set. Better results are obtained from solutions that are more sophisticated, like greedy-based selection, along with extensions it may have, however, the computational costs are substantial and it is not easily implemented on networks that are very complex [26].

Structural measures are used to improve optimization, so that nodes in the same network segments are not selected to allow a better seed allocation. These types of solutions are based more on better use of processes of natural diffusion and use sequential seeding [27], avoid nodes from within the same communities with intra connections that are close by using target communities [28], use dynamic rankings with sequential seeding [29] and use mechanisms for voting that have lower weights once activated nodes have been detected [30]. Apart from basic centrality measures, the central nodes in networks can be detected using a k-shell based approach [31]. Alternative approaches use bio-inspired algorithms to select the initial set of nodes [32].

The majority of earlier approaches are based on networks that are static, while the more recent studies account for networks that are dynamic. They have temporal characteristics and more reality-based specifics, as opposed to static snapshots [17]. The other research paths took base on the multi-layer networks and processes of information spreading that are intra layer [16]. There have been attempts to use other knowledge about the on-going processes to obtain better results by using adaptive seeding, even though the majority of solutions are geared to the seed set initiated processes [33]. Other approaches account for numerous campaigns that are on-going, as well as how they interrelate [34].

Unfortunately, the knowledge about the social network in which the campaign is going to be performed is often limited to some basic characteristics. Moreover, the number of nodes and edges in a real social network is often immense, which makes advanced simulations infeasible. The research takes into account simulations within synthetic networks to investigate phenomena within different network structures. While collecting information about real networks is difficult, synthetic networks based on theoretical models can be used. Moreover, the structure of synthetic networks can be adjusted during the generation process, thus allowing the researchers to perform a more profound analysis of the processes in complex networks. Simulation studies often use networks based on the free-scale model proposed by Barabasi-Albert (BA) [35], small world model proposed by Watts-Strogatz (WS) [36] and random graph model introduced by Erdos-Renyi (ER) [37]. For example [38] used WS, ER and BA synthetic networks for modeling interacting processes, [12] analyzed the role of structures of ER, BA, and WS networks on campaign performance, [39] proposed a framework to analyze multiple spreading processes and verified it with the use of BA networks, [40] used WS networks for cooperative epidemic modeling.

**Fig 1:**
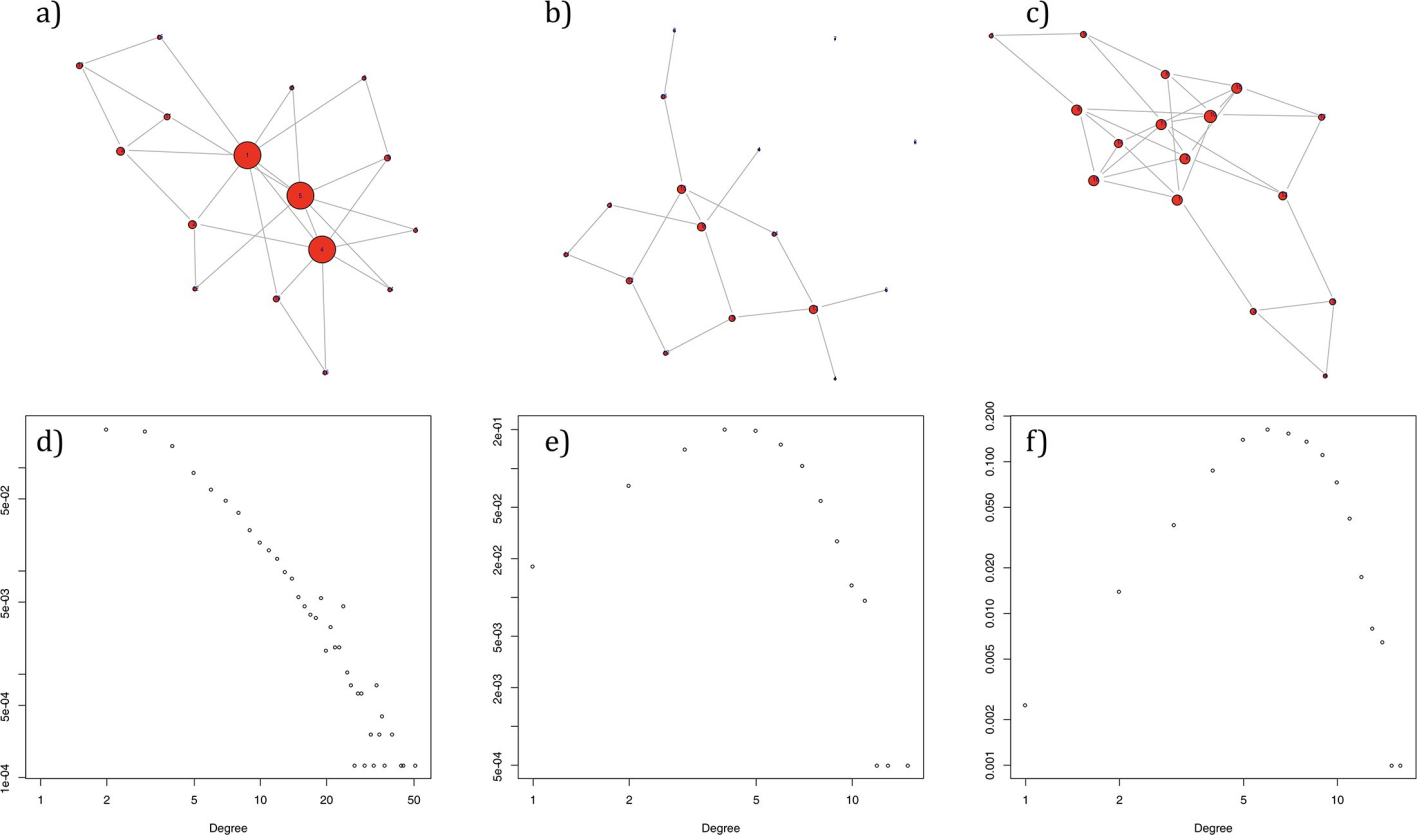
Graph representation of example 16-node synthetic networks: a) BA, b) ER, c) WS; and degree distribution charts of example 2000-node synthetic networks: d) BA, e) ER, f) WS.

The characteristics BA and WS theoretical models are close to real systems. The Barabasi-Albert network model was first created in 1999, as a result of a study of the at-the-time structure of the WWW. The construction of BA networks is based on two complementary mechanisms: network growth and the mechanism of preferential attachment. The BA model is similar to several natural and human-made systems, such as the Internet, WWW, citation networks or social networks, to name just a few, where several selected nodes (hubs) have unusually high degree compared to the remaining nodes of the network. Fig 1a presents an example of a Barabasi-Albert network and the chart on Fig 1d depicts the degree distribution of a sample BA model. The Erdos-Renyi network model was first described in 1959 and is constructed on the assumptions that at first the number N of nodes is defined and, subsequently, from all $\left(\begin{array}{c} N\\ 2 \end{array}\right)$ pairs of nodes, random E pairs are selected between which the edges are created. A sample ER model and degree distribution of a sample ER model is presented on Fig 1b and 1e respectively. The ER model offers a simple and powerful model with many applications, but might be inappropriate for modeling some real-life phenomena, due to the fact it does not generate local clustering of nodes. Therefore, in 1998 the Watts-Strogatz model was created to addresses this issue. The WS model accounts for clustering, but keeps the short average path lengths from the ER model. Fig 1c presents an example of a WS network and the chart on Fig 1f depicts the degree distribution of a sample WS model.

### 2.2 MCDA foundations of the proposed approach

In case of a viral marketing campaign in a social network, the ordering party might be interested not only in maximizing the coverage of the campaign, but also in affecting its dynamics, as well as keeping the campaign cost within a reasonable budget. All these aspects need to be considered before launching the campaign. Therefore, planning a viral marketing campaign in social network is a multi-criteria problem, which can be presented as (2) [41]:

$$\begin{array}{c} max\;\{c_{1}\left(a\right),c_{2}\left(a\right),\ldots ,c_{k}\left(a\right)|a\in A\}, \end{array}$$ (2)

where *A* is a finite set of possible campaign strategies {*a*_1_, *a*_2_, …, *a*_*n*_}, whereas {*c*_1_(⋅), *c*_2_(⋅), …, *c*_*k*_(⋅)} is a set of evaluation criteria. Some of the criteria might be maximized and others minimized. The criterial performance of each strategy regarding each criterion can be expressed in a form of a performance table. Intuitively, it is expected from the decision maker (DM) to identify the strategy that optimizes all criteria. However, usually there exists no alternative that optimizes all criteria simultaneously.

Let us consider an example viral marketing campaign, for which multiple alternative strategies were prepared. The strategies are characterized by three criteria: seeding fraction, propagation probability and the potential coverage that can be obtained. The coverage is a very important criterion, however, generally a strategy that obtains 100% coverage is not always chosen, as it would require infecting a massive number of initial seeds in the network or providing multiple incentives to increase the propagation probability in the network. On the other hand, if a strategy with minimal seeding fraction and minimal propagation probability is chosen, it cannot be expected to cover the complete network. Therefore, a compromise solution between the strategies should be chosen.

It is important to note that the solution to a multi-criteria problem depends not only on the criterial performance of each alternative, but also on the campaign ordering party itself. There is no absolute best strategy for all campaigns and the best compromise strategy depends on the preferences of the DM.

Three natural dominance relations can be associated to a decision problem of the multi-criteria nature presented in (2): indifference, preference and incomparability. Let us consider two alternatives *a* and *b*. If for every criterion *c*_*i*_ *a* is as good as *b*, then the two strategies are indifferent (*aIb*). If for every criterion *c*_*j*_ *a* is as good or equal to *b* and there exists at least a single criterion *c*_*k*_ for which *a* is better than *b*, then *a* is preferred to *b* (*aPb*).

Finally, if there is a criterion *c*_*m*_ for which *a* is better than *b*, but there also exists a criterion *c*_*n*_ for which *b* is better than *a*, then the two strategies are incomparable (*aRb*). Strategies which are best at each criterion are rare and, therefore, usually most strategies are incomparable without additional information from the campaign ordering party. This information can include inter alia the weights expressing the relative importance of each criterion or preferences associated to each pairwise comparison of strategies when each criterion is considered on its own [41].

Multiple multi-criteria decision analysis methods have been invented in order to reduce the number of incomparabilities (*R*) in the decision graph between the considered viral marketing campaign strategies. The MCDA attempts to handle this task can be generally divided into two approaches, the so-called American and European MCDA schools. The former is based on aggregating all the decision-making problem criteria into a single criterion—a utility function. Such approach has the benefit of providing the possibility to produce a complete ranking of strategies with a precise score given to each one. However, such approach largely transforms the structure of the decision problem. On the other hand, Roy [42, 43] proposed to construct outranking relations by enriching the dominance relation between the alternatives where possible. In such an approach (European MCDA school), not all incomparabilities are eliminated, however, a reliable selection of the best alternative is possible.

Presently, the literature review allows to observe a number of approaches (MCDA methods) based on the above American and European approaches [44]. Discussions about the up-to-date MCDA methods can be found inter alia in [45, 46]. The AHP, TOPSIS and Electre methods are often indicated as popular and widely used in the problems of evaluation and ranking creation [47, 48]. The selection of the aggregation technique (the utilized MCDA method) may influence the quality of the obtained modeling results [49–52] and requires justification in the context of the modeling aspects adapted in the paper [53, 54].

When analyzing the characteristics of the data and the environment of the constructed MCDA model, it should be noted that the input data of the model has a quantitative character and is expressed on the cardinal scale. It was decided that the weights of the individual criteria should be taken into account and that they should be expressed using explicitly specified numerical values [55]. Thus, the result is expected to be expressed on a quantitative scale [53]. The modeling process also assumed the natural imprecision of the preference information, which in practice, in MCDA methods, takes the form of complex preferential functions (e.g. pseudo-criteria) [42]. Additionally, the construction and usage (decision problematic) of the model, should result in a ranking of variants (strategies) [43]. The obtained ranking is expected to provide a complete order of the strategies [56]. None of the popular MCDA methods (AHP, TOPSIS, Electre) meet all of the indicated requirements at once. Based on a set of 56 MCDA methods discussed in [45] and the MCDA taxonomy contained there, it is easy to show that these assumptions are fully implemented only by the PROMETHEE II method. Therefore, it was decided to use this method in the next steps of the data analysis.

PROMETHEE is a family of MCDA methods that use pairwise comparison and outranking flows to produce a ranking of best decision variants [57]. The weights expressing the relative importance of each criterion need to be specified by the decision maker. This is a complicated process based on the DM’s priorities and perceptions. The actual values of the criteria weights can be freely selected by the campaign ordering party. Fortunately, multi-criteria decision analysis provides tools such as sensitivity and robustness analyses, which allow to verify the effects the chosen values have on the resulting rankings and to sequentially adjust the weights.

**Fig 2:**
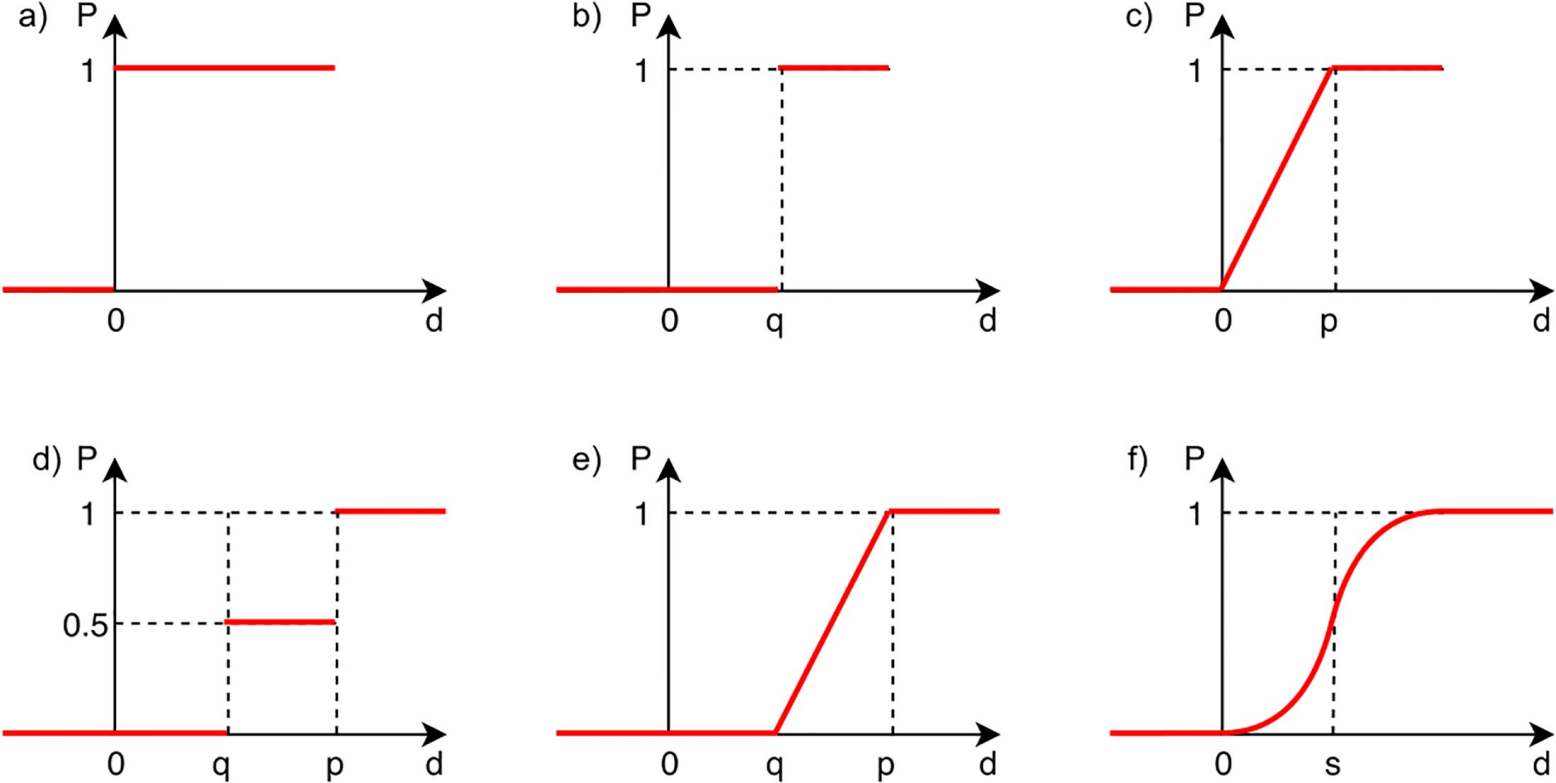
Visual representation of the six preference functions used in the PROMETHEE methods: a) usual criterion, b) U-shape criterion, c) V-shape criterion, d) level criterion, e) V-shape with indifference criterion, f) Gaussian criterion.

When the PROMETHEE methods are used for viral marketing campaign strategy selection, all strategies are compared pairwise. The preference for one alternative over another is studied under each criterion. For a small difference *d* in evaluations of the two compared strategies, a small preference *P* would be assigned to the better one, or no preference at all, if the difference is negligible. On the other hand, the larger the difference *d* between alternatives, the higher the preference *P*. The preference *P* between strategies *a* and *b* under criterion *c*_*j*_ is expressed as real numbers *P*_*j*_(*a*, *b*) and is in the range between 0 and 1. The actual preference value assigned depends on the preference function in the DM’s brain. The authors of the PROMETHEE methods propose six preference functions to express the preference function of the DM: usual criterion, U-shape criterion, V-shape criterion, level criterion, V-shape with indifference criterion, Gaussian criterion (see Fig 2 and Table 1) [41, 43, 58]. The three variables presented in Table 1, i.e. *p*, *q* and *s* require an explanation. The *p* preference threshold is the smallest difference between two alternatives that would result in a full preference of one alternative over the other. The *q* indifference threshold is the largest difference between two alternatives that the DM considers negligible. Parameter *s* denotes the inflection point of the Gaussian preference function and should be selected as a value between *q* and *p*.

**Table 1:** Formulae for the six preference functions used in the PROMETHEE methods [41].

**a) usual**	**b) U-shape**	**c) V-shape**
$P\left(d\right)=\{\begin{array}{cc} 0 & d\leq 0\\ 1 & d>0 \end{array}$	$P\left(d\right)=\{\begin{array}{cc} 0 & d\leq q\\ 1 & d>q \end{array}$	$P\left(d\right)=\{\begin{array}{cc} 0 & d\leq 0\\ \frac{d}{p} & 0\leq d\leq p\\ 1 & d>p \end{array}$
**d) level**	**e) V-shape with *q***	**f) Gaussian**
$P\left(d\right)=\{\begin{array}{cc} 0 & d\leq 0\\ \frac{1}{2} & q\leq d\leq p\\ 1 & d>p \end{array}$	$P\left(d\right)=\{\begin{array}{cc} 0 & d\leq 0\\ \frac{d-q}{p-q} & q\leq d\leq p\\ 1 & d>p \end{array}$	$P\left(d\right)=\{\begin{array}{cc} 0 & d\leq 0\\ 1-e^{-\frac{d^{2}}{2s^{2}}} & d>0 \end{array}$

It was decided in the process of preference modeling to use two of the six preference functions: V-shape and Gaussian. Their choice was dictated by the possibility of including the natural imprecision of the preference information of the decision maker into the modeling process [41, 43]. The form of the V-shape function, being the most complex structure of the preference function, is based on the concepts of strong and weak preferences, as well as indifference [43]. It is directly related to the concept of a pseudo-criterion, i.e. criterial function with the thresholds of indifference and weak and strong preference. This, in contrast to pre-criterion, quasi-criterion and real criterion, allows to effectively model the areas of information uncertainty of the decision-maker and, consequently, also to examine the robustness of the model in a wider scope, instead of simply generating a ranking [53]. Complementary, for comparative purposes, the Gaussian form was used as the second preferential function. In this research, contrary to the classic tasks of the MCDA methods, where only a small number of variants is being ordered [58], the obtained sample is fairly complex. It was assumed that the distribution of preferences is based on the Gauss function and, thus, reflects the normal distribution. Such representation allows to build, based on the given sample, a softer form of the preferential function (when compared to the linear form of the V-shape function). It is worth noting that this form of the preference function is based exclusively on the concepts of weak preference and indifference.

For each viral marketing campaign strategy, an aggregated preference index can be computed with the formula (3):

$$\begin{array}{c} \left\{ \begin{array}{c} \pi \left(a,b\right)=\sum_{j=1}^{k}P_{j}\left(a,b\right)w_{j}\\ \pi \left(b,a\right)=\sum_{j=1}^{k}P_{j}\left(b,a\right)w_{j} \end{array}\right. \end{array}$$ (3)

where *w*_*j*_ denotes the weight assigned to the *C*_*j*_ criterion. *π*(*a*, *b*) ∼ 0 implies a weak global preference, whereas *π*(*a*, *b*) ∼ 1 implies a strong global preference of *a* over *b*.

The obtained indices are then used to calculate the positive and negative outranking flows with (4) and (5) [41]:

$$\begin{array}{c} \phi^{\;+}\left(a\right)=\frac{1}{n-1}\sum_{x\in A}\pi \left(a,x\right) \end{array}$$ (4)

$$\begin{array}{c} \phi^{\;-}\left(a\right)=\frac{1}{n-1}\sum_{x\in A}\pi \left(x,a\right) \end{array}$$ (5)

The *ϕ*^+^(*a*) value indicates the *strength* of alternative *a*, i.e. how well it is outranking other alternatives. On the other hand, the *ϕ*^−^(*a*) value represents how the alternative *a* is outranked by other alternatives, thus showing its *weakness*. The PROMETHEE I method uses the *ϕ*^+^ and *ϕ*^−^ values to produce a partial ranking of the alternatives [41]. The usage of the PROMEHTEE II method, in turn, would allow to obtain the complete ranking of the campaign strategies based on the net outranking flow (6):

$$\begin{array}{c} \phi \left(a\right)=\phi^{\;+}\left(a\right)-\phi^{\;-}\left(a\right) \end{array}$$ (6)

For two strategies *a* and *b*, if *ϕ*(*a*) > *ϕ*(*b*) then *aPb*. Contrarily, if *ϕ*(*a*) = *ϕ*(*b*) then *aIb*.

If the criterion *c*_*j*_ is given the weight of 100% while the rest of the criteria is given the weight of 0%, a single criterion net flow for each strategy *a* is obtained: *ϕ*_*j*_(*a*). When all single criterion net flows for all *k* criteria and *n* strategies are known, then all strategies can be represented as points in a *k*-dimensional space. Since the decision problems usually consist of more than two criteria, the *n* points from the *k*-dimensional space need to be projected to a plane.

The family of PROMETHEE methods comes with the GAIA visual modeling. A GAIA plane is a plane on which the alternatives and the criteria unit vectors are projected, for which as much information as possible is preserved after projection. The quantity of information that was preserved by the projection is denoted as *δ*. The GAIA plane can successfully support the decision problem analysis if *δ* >= 50%. The GAIA plane allows the analyst to learn the information about the criteria and alternatives in a decision problem [41]:

- the length of the criterion vector on the plane represents how discriminating the criterion is. The longer the vector, the more effect the criterion has on the final decision;
- the vectors of criteria similar in terms of preference are pointed in similar directions;
- the vectors of criteria conflicting in terms of preference are pointed in opposite directions;
- the vectors of criteria not related to each other in terms of preference are pointed orthogonally;
- alternatives with similar performance are grouped closely together;
- alternatives supported by a particular criterion are located in the direction pointed by this criterion’s vector.

**Fig 3:**
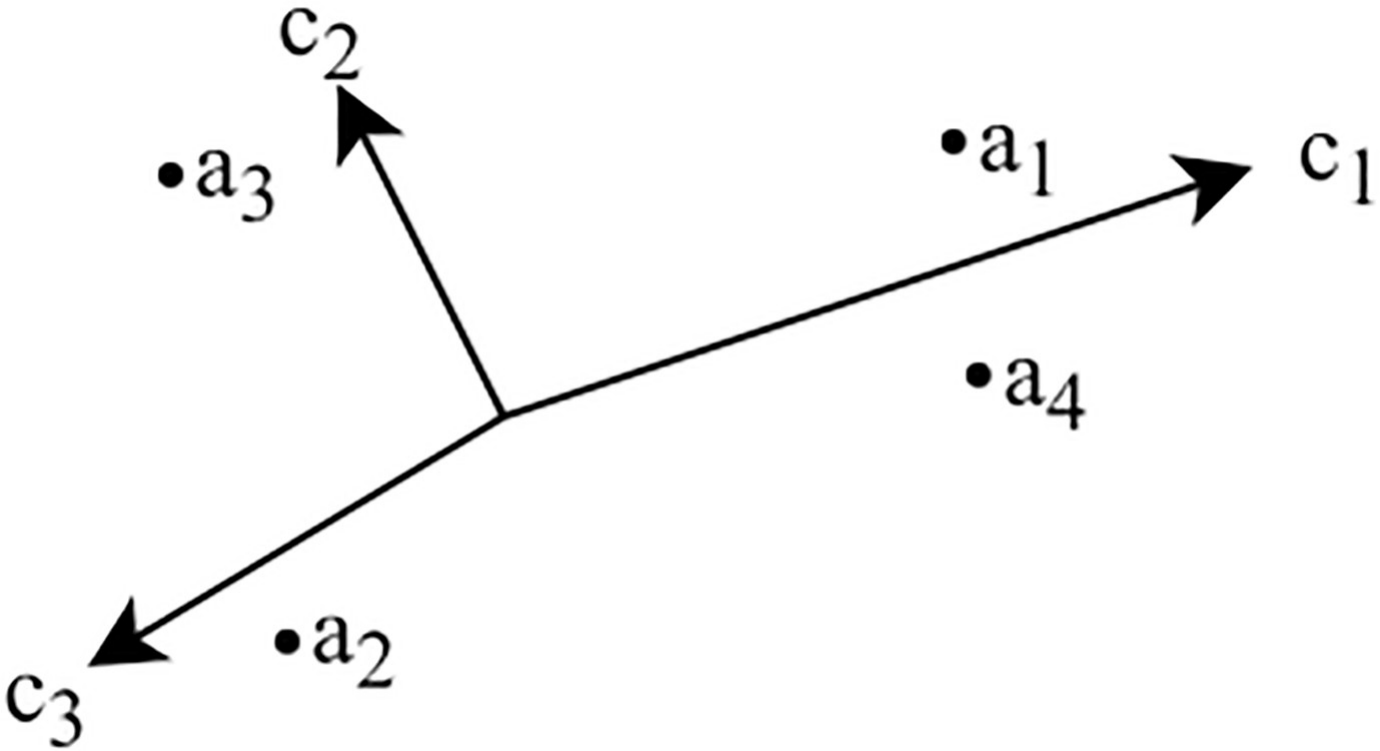
Example GAIA plane representing a viral marketing campaign strategy selection with four possible strategies and three criteria: *c*_1_—Maximization of coverage, *c*_2_—Minimization of the number of iterations, *c*_3_—Minimization of the seeding fraction.

An example GAIA plane for a sample viral marketing strategy campaign selection problem with four possible strategies and three criteria (*c*_1_—maximization of coverage, *c*_2_—minimization of the number of iterations, *c*_3_—minimization of the seeding fraction) is presented on Fig 3. The analysis of the example allows to observe that, since the *c*_1_ and *c*_3_ vectors are pointing in opposite directions, the preference for maximizing the coverage is in conflict with the preference for minimizing the seeding fraction. On the other hand, the *c*_2_’s orthogonal direction compared to *c*_1_ and *c*_3_ implies that the preference for minimization the number of iterations is not related to the preference for maximizing the coverage or minimizing the seeding fraction. The lengths of the criteria’s vectors suggest that the maximization of coverage (*c*_1_) is most discriminating in this decision problem. The alternatives *a*_1_ and *a*_4_ are grouped together at the location pointed by the vector *c*_1_, therefore, it can be assumed that they both are similar to each other and mostly supported by the coverage maximization criterion. On the other hand, they are located in a direction opposite to the direction of the *c*_3_ vector, which implies that they fail to use a small seeding fraction.

### 2.3 Conceptual framework and evaluation criteria

**Fig 4:**
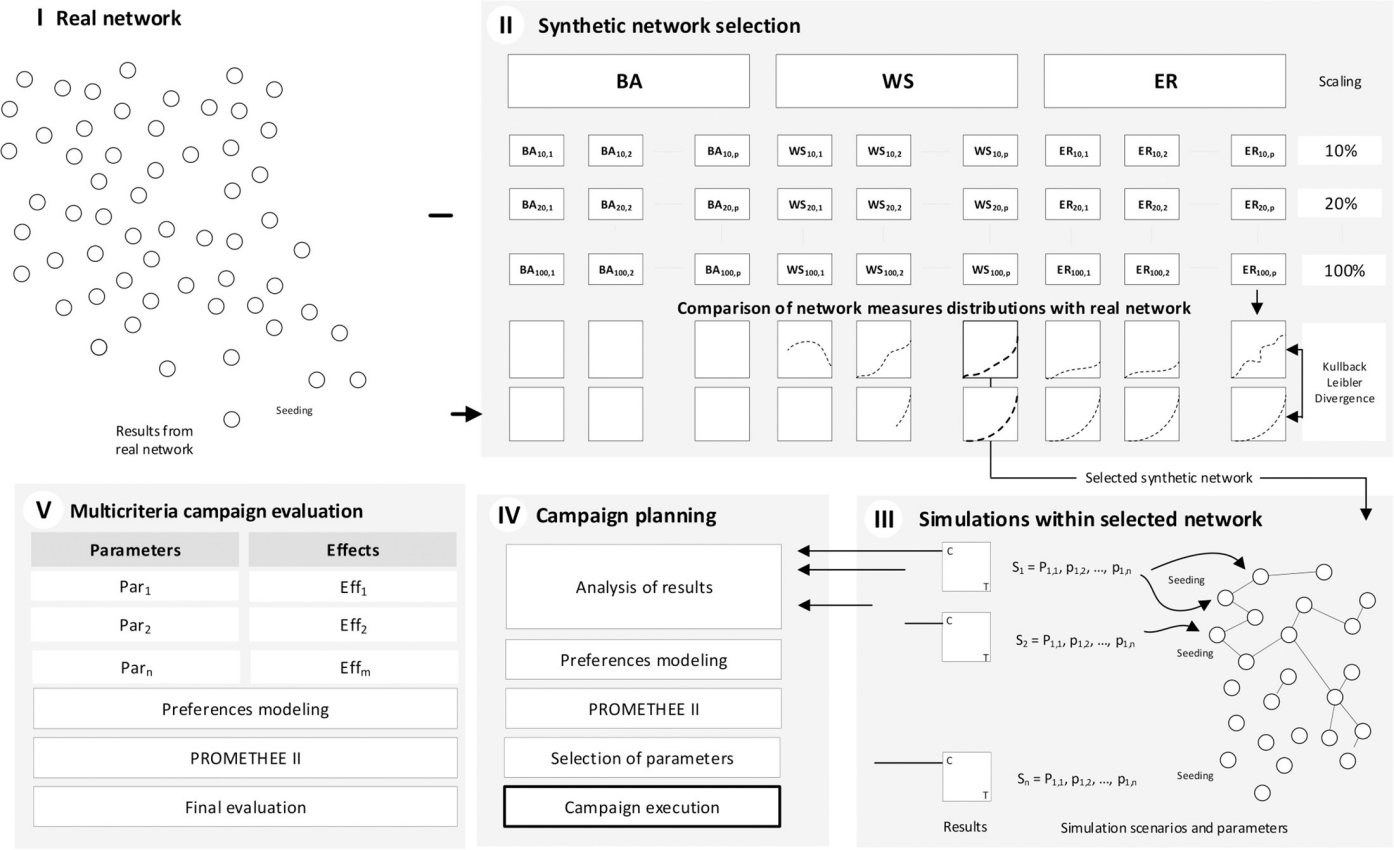
Proposed framework based on five stages: Analysis of real network (I), synthetic network selection process (II), simulations within synthetic network (III), campaign planing (IV) and multi-criteria campaign evaluation(V).

The selection of the best strategy for a viral marketing campaign in social networks is a complex decision-making problem based on multiple criteria. Moreover, running simulations on a real network is most often time consuming and sometimes impossible. Therefore, in the presented approach (see Fig 4) the authors propose to run the planning process on a synthetic model, which has similar properties to the target real network, yet allows to perform multiple simulations resulting in data for the performance table as an input to the strategy evaluation process.

In order to obtain such a synthetic model resembling the target real network, the authors propose to generate a set of BA, ER and WS networks with various parameters and number of nodes equal to 10%, 20%, … 100% of the real network. Subsequently, KullbackÔÇôLeibler divergence [59] can be used to learn which of the generated networks is closest to the real one. Criteria affecting the complexity of simulations, such as number of nodes and edges, can also be considered. Additional criteria can also be added to the decision process, depending on the analyst’s needs. When the performance table is created for all synthetic networks and all criteria, the most preferred one should be selected based on the gathered data with the use of MCDA methods.

The next element of the proposed framework is the decision model structuring process, during which the decision criteria for evaluating the possible campaign strategies are chosen. In the authors’ approach, the criteria can be divided into two groups. The first group contains the input criteria for constructing a strategy—Par1, Par2, …, Par*m*. The second group contains the strategy performance evaluation criteria Eff1, Eff2, …, Eff*n*, whose values are based on achieved effects and can be obtained by simulating each strategy. Nevertheless, the proposed framework assumes the decision-maker’s freedom in selection of the decision criteria and in grouping them into clusters, depending on the campaign ordering party requirements.

After the criteria have been selected and the decision model has been structured, the chosen synthetic network should be used to perform a complete set of simulations required to obtain the performance table containing the evaluations of each strategy. For simulations independent cascades model (IC) [26] was implemented. Information spreading process is initiated by a set of nodes activated by seeding. Spreading is based on propagation probability *PP*(*a*, *b*) that node *a* activates node *b* in the step *t* + 1 under condition that node *a* was activated at time *t* by other node or was selected as a seed [60]. The main reason for selecting this model was a relatively small number of seeds needed to induce diffusion what can be important for small networks. In linear threshold model (LT) small number of activated nodes would have no effect [26].

Subsequently, the obtained performance table is used to perform MCDA analysis of the possible strategies with the use of PROMETHEE II. The analysis includes the following aspects:

- generation of a complete ranking of the viral marketing campaign strategies, based on various preference functions;
- usage of the GAIA plane to verify how each criterion affects the strategy selection;
- performing sensitivity analysis to verify the stability intervals of the rankings of the leading campaign strategies.

It should be noted that during the analysis, the preference modeling step is repeated multiple times. The initial preference weights of the criteria can be subsequently modified to verify the robustness of the obtained strategy selection problem solution. Eventually, the analyst provides the recommendation which strategy, i.e. campaign parameters, should be used to run the campaign on the target real network. Last, but not least, the authors’ proposed framework can also be used to monitor the results of the executed campaign, as well as to perform a multi-criteria evaluation of the campaign strategies on a real network.

The conceptual framework proposes a model in a generalized form with Par1,Par2,…,Par*m* criteria related to campaign settings treated as input variables and Eff1,Eff2,…,Eff*n* evaluation metrics related to campaigns results understood as output variables. The generalized model can be parametrized for campaigns with different mechanics, used strategies and goals. Like it was discussed in [19], criteria can vary across different campaigns, sectors, strategies and available resources. Therefore, a set of multi-criteria decisions needs to be made at the planning stage of the viral marketing campaign, based on the campaign objectives and available budget. It leads us to framework verification with the use of parameters mapping initial viral campaigns parameters and settings into the parameters of the simulation model. For the model validation we propose a set of criteria discussed in earlier studies and the specifics of the used simulation with the independent cascade model and the agent based approach presented in Table 2.

**Table 2:** Mapping viral campaign characteristics into simulation model parameters and outputs.

Criteria	Type	Viral campaign	Model parameter	Symbol
Par1	Input	Number of initial customers	Percentage of network nodes activated during seeding process (seeding fraction)	SF
Par2	Input	Motivation to spread the content	Propagation probability	PP
Par3	Input	Initial customers selection	Computing node rankings and selection of nodes with highest propagation potential	R
Eff4	Output	Time required to reach assumed number of customers	Number of simulation steps	S
Eff5	Output	Number of reached customers	Number of activated nodes within the network	C

#### 2.3.1 The number of initial customers and seeding fraction (Par1)

The process of information spreading in viral marketing campaign in social networks is initialized with seeding the advertising content to a group of people (initial set of nodes). The fraction of nodes that are selected from the network for seeding can be adjusted according to the campaign objectives and it is affecting the dynamics and coverage of the process. The earlier research usually uses fixed ranges of seeding percentage as a parameter [7]. The activation of the initial seeds is recognized as the main cost of viral marketing campaigns [61]. The cost can be growing for highly influential nodes, while they attract high attention from marketers and the users from their direct and indirect connections. Other research focuses on minimization of the seed set to reduce the initial costs with probabilistic coverage guarantee [62]. The cost-effectiveness can decrease when more nodes are added to the seeding [63]. If too many users are targeted, an overexposure effect takes place [64]. While the activation of large fraction of network nodes as the seed set can result in high number of nodes reached within the network, it requires high activation costs. The goal can be to use the smallest possible seed set delivering satisfactory results [65] [62]. To include the above factors, we define criterion Par1 representing the fraction of nodes used as the initial seeds denoted as seeding fraction (SF) within the simulation model.

#### 2.3.2 Spreading the content and propagation probability (Par2)

In order to motivate the network members to pass the information further, some financial investments need to be made. As a result, the propagation probability is directly related to the campaign costs. From the practical point of view, the propagation probability can be increased with coupons and other incentives [66]. Authors discuss the role of incentives for increasing the camping dynamics and the costs of incentives is related to the degree of the target nodes [67]. The proposed approach minimizes the cost while guarantees the number of reached users. One of the strategies is enforcing the propagation dynamics without the use of additional seeds and users with high centrality measures which are expensive to reach [61]. Activation of early adopters and increasing their propagation probabilities may require higher incentives [68]. Multi-scale incentives can be used for users from different target groups to further boost the diffusion rate [69]. The top influential nodes, such as a popular user, may require more incentives to be recruited as a Seed [70]. To generalize the aforementioned factors, we use Par2 as the main result of the increased motivation and propagation probability (PP) during the simulations.

#### 2.3.3 Selection of initial customers and nodes ranking method (Par3)

The nodes for seeding are selected based on their ranks computed from various centrality measures, such as degree, betweenness, closeness or eigenvector centrality. Each centrality measure requires some level of effort, indirectly related to a third kind of cost. Intuitively, if the seeding fraction is high and the network members are motivated to increase their propagation probability, the process of information diffusion should execute dynamically and achieve high network coverage. However, the budget for the campaign can be limited. Moreover, the aim of the campaign might not be to achieve high coverage very fast, but to keep the campaign slowly crawling for a longer amount of time. Computational cost of choosing seeds was analyzed earlier in relation to greedy algorithm [71]. Another study discusses computational costs and propose upper-bound estimation based algorithm to accelerate the computing speed [72]. Authors with the same approximation ratio like greedy algorithm [26]. The authors of [73] emphasize that the earlier approaches use impractical assumptions that any seed user can be acquired with the same cost and the same is the benefit obtained when influencing each user. Study [73] proposes cost-aware targeted viral marketing focused mainly on of selecting a node. Costs may represent the degree of difficulty with which people accept specific information [74]. From the perspective of network analysis, the centrality measures like page rank can represent costs because they are usually proportional to social influence. They can be used for mapping the corresponding cost values to all users in a given social network. Positive correlation between degree centrality and the success of viral marketing is observed [7]. To include above factors within the model, we assume different costs for different rankings methods. Nodes selection costs are represented by parameter Par3 within the model. For example they are lower for degree and higher for betweenness computations during simulations.

#### 2.3.4 Campaign duration and number of simulation steps (Eff4)

While Par1-Par3 are related to the model inputs and key factors affecting campaign performance like number of initial customers, budges, incentives and other forms of customer motivation, the proposed approach assumes monitoring of the campaign effects and assigning to them the preferences of the decision maker. The campaign cognitive goals can be based on reach, awareness and knowledge, behavioral goals are represented by number of actions and rate at which creatives are transferred [19]. From the perspective of the decision maker, the time when the assumed number of messages is received can be crucial. One of the goals can be minimizing the time in which assumed coverage is achieved [75]. Other authors emphasize the velocity and the speed of transmission, persistence and mental barriers [20]. Another study minimizes the complete influence time with cost represented by a fuzzy variable [21]. The role of time was emphasized in terms of campaigns with limited time (eg. political campaigns) [76]. In the proposed model, the duration of the campaign is represented by evaluation criteria Eff4 and (in simulations) as the number of simulation steps until the process is finished.

#### 2.3.5 Campaign coverage and the total number of activated nodes (Eff5)

Another measured result is related to the network coverage and is represented by criterion five (Eff5). It is the most common effect taken into consideration. Most of research focuses on maximizing reach and number of infected nodes and is treated as the main goal of a campaign [18]. The ability to reach a large number of customers with limited advertising budget is the key feature of the viral marketing [2]. From the perspective of algorithms, total coverage is the key evaluation factor used for influence maximization problem and seed selection algorithms evaluation [26]. It is represented by the number of activated nodes within the network used during simulations.

## 3 Planning a viral marketing campaign with the use of synthetic networks

The empirical study has been divided into two subsections—planning and evaluation. In the former, a substantially smaller synthetic network was chosen to facilitate the planning of a viral marketing campaign. In the latter, an evaluation of marketing strategies in a real network [77] was presented.

### 3.1 Synthetic network selection

**Fig 5:**
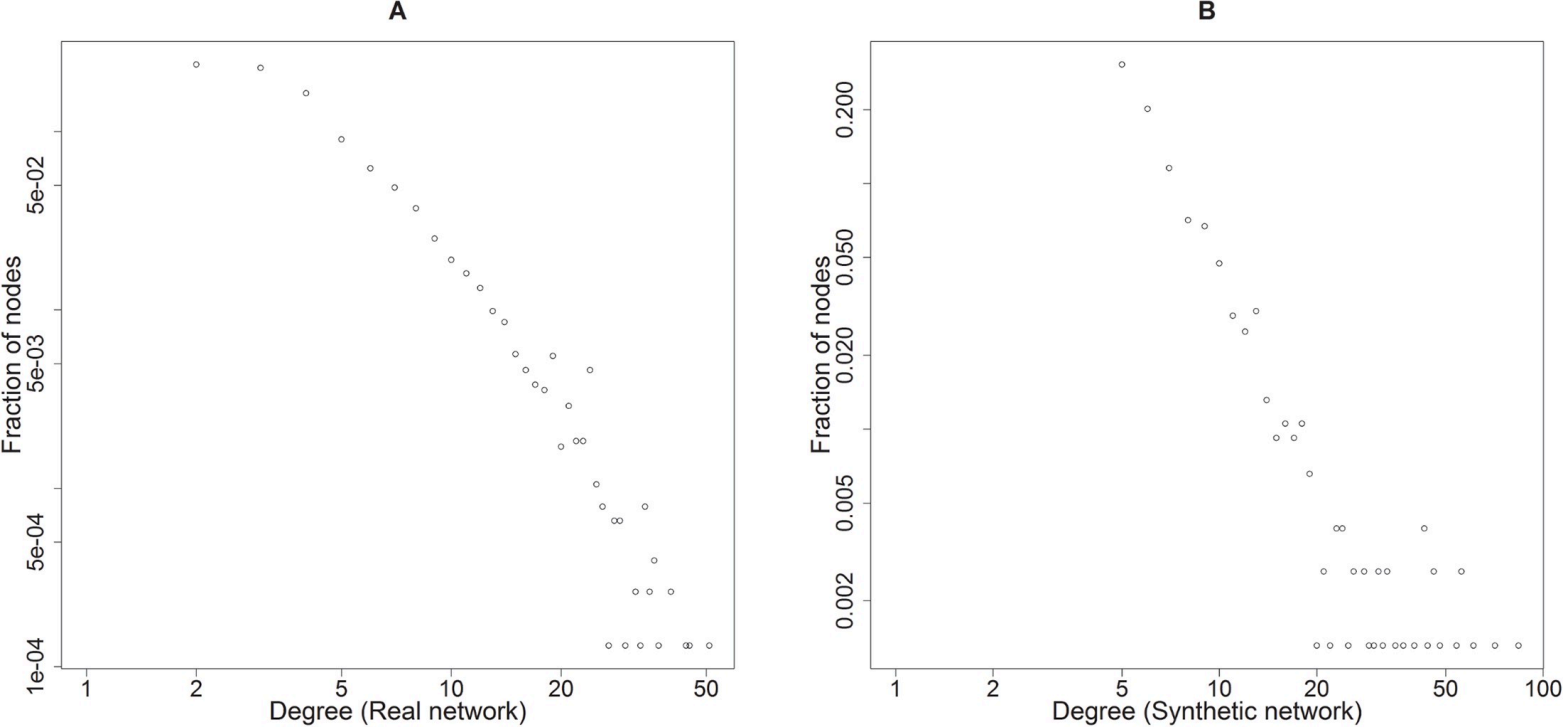
Degree distribution chart of A) the real network, B) the selected synthetic network.

In the empirical research, the authors used the proposed framework to plan a viral marketing campaign for a real network [77]. The real network is based on 7610 nodes, 15751 edges with average values of main metrics: total degree D = 4.14, closeness C = 0.0004, PageRank PR = 0.0001, EigenVector EV = 0.003, clustering coefficient CC = 0.49 and betweenness B = 13478.93. The degree distribution of the network is presented on the chart on Fig 5a. In order to approximate the real network, a set of 150 synthetic networks was generated. This set was built by combining three network models (BA, ER and WS) with the following parameters: percentage of nodes of the real network—10%, 20%, …, 100%, i.e. 761, 1522, …, 7610 nodes; out-degree parameters with values 1,2,3,4 and 5 for the BA networks; number of edges in graph equal to 100%, 200%, 300%, 400% and 500% for the ER networks and neighborhood within which the vertices of the lattice will be connected with values 1,2,3,4,5 with rewiring probability 0.5 for the WS networks.

**Fig 6:**
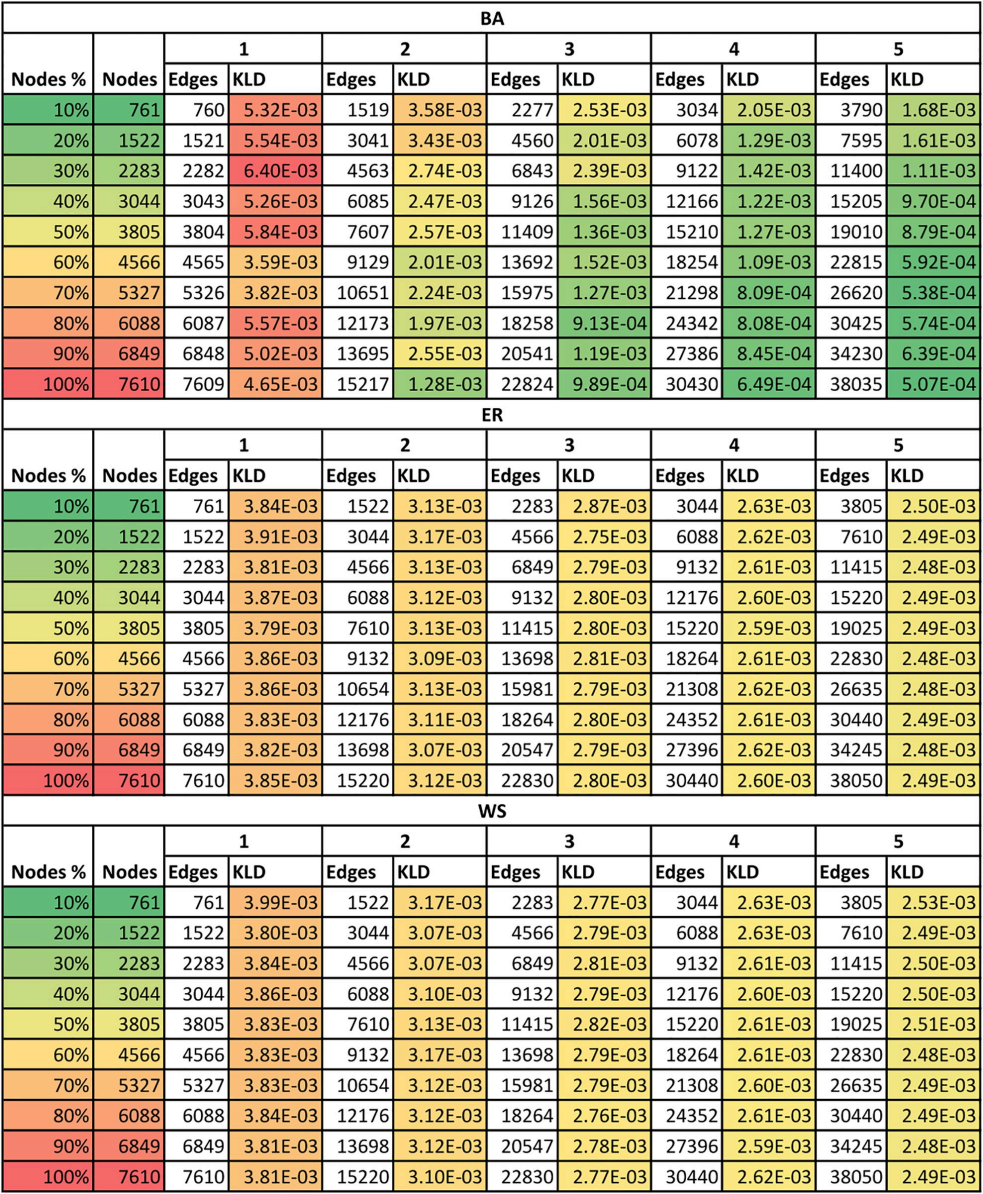
Visual representation of the 150 synthetic networks used to approximate the [77] real network.

Kullback-Leibler divergence (KLD) [59] was used to evaluate the similarity of the synthetic networks to the real network. The results are visually presented on Fig 6. The analysis of Fig 6 allows to observe that the ER and WS synthetic networks are moderately similar to the real network, regardless of the size of the network or parameters selected. On the other hand, in case of the BA networks the similarity to the real network depends on the number of nodes and parameters chosen. The more nodes and edges, the closer its degree distribution is to the degree distribution of the real network. The lowest KLD value was observed for the BA network with 7610 nodes and 38035 edges. However, selection of such a network provides little computational benefit compared to the original real network. Therefore, the actual network for the campaign planning was selected with the use of MCDA analysis of all the 150 potential synthetic networks based on the following criteria: K1—number of nodes, K2—number of edges and K3—KLD value; with the preference of the minimum values for all criteria and equal weights of all the criteria. As the result of the analysis, the BA network containing 10% nodes of the original network (761) and 3034 edges with network metrics degree D = 7.97, closeness C = 0.3211, PageRank PR = 0.0013, EigenVector EV = 0.086, clustering coefficient CC = 0.040 and betweenness B = 812.03 was selected. The degree distribution of the selected synthetic network is presented on the chart on Fig 5b.

### 3.2 Overview of the simulations in the synthetic network

In order to ascertain repeatability of the results regardless of the simulated parameters, ten simulation scenarios were generated, in which for each node a random value from the range of < 0, 1 > was assigned. This value was later used in the simulations to decide if the particular node passes the information through (the drawn value was smaller than the simulated propagation probability) or if the propagation stops (the drawn value was higher than the simulated propagation probability).

During the simulation stage, a total of 400 sets of parameters was tested, as a Cartesian product of the following parameter values:

- Par1—0.01, 0.02, 0.03, 0.04, 0.05, 0.06, 0.07, 0.08, 0.09, 0.10
- Par2—0.01, 0.10, 0.20, 0.30, 0.40, 0.50, 0.60, 0.70, 0.80, 0.90
- Par3—degree [0.0060], betweenness [0.0110], closeness [0.0085], eigenvector centrality [0.0090]—the values assigned to each measure were obtained based on the actual time of ranking generation based on each measure.

Each simulation was repeated for all 10 scenarios, thus resulting in 4000 simulation runs. After each simulation run, the iteration of the last infection, as well as the achieved coverage was registered. Their averaged values were then saved as the empirically measured performance values of the Eff4 and Eff5 criteria.

### 3.3 PROMETHEE II analysis

After the simulations finished, the output from subsection 3.2 was used to create performance tables for the PROMETHEE II analysis. Initially, a V-shape preference function was used to model the comparison preferences, with the indifference threshold *q* = 0 (no uncertainty taken into consideration) and preference threshold *p* equal to the standard deviation value for each criterion Par1-Eff5. The preference direction for the cost criteria Par1-Par3 were minimized and for the dynamics and coverage criteria Eff4-Eff5 maximized. Initially, every criterion was assumed to be equally important and, therefore, the weights of all criteria were set to 1 (see Table 3a).

**Table 3:** PROMETHEE II parameters for the synthetic network.

	Par1	Par2	Par3	Eff4	Eff5
	**Statistics**				
Minimum	0.01	0.01	0.0010	1	1.05%
Maximum	0.1	0.9	0.0110	10.40	100%
Average	0.06	0.45	0.0086	5.19	75.91%
Standard Dev.	0.03	0.29	0.0019	2.09	32.76%
**a)**	**V-shape, q = 0**				
Q: indifference	0	0	0	0	0
P: preference	0.03	0.29	0.0019	2.09	32.76%
weights	1	1	1	1	1
**b)**	**V-shape, q = 50% SD**				
Q: indifference	0.015	0.145	0.0009	1.045	16.38%
P: preference	0.03	0.29	0.0018	2.090	32.76%
weights	1	1	1	1	1
**c)**	**Gaussian**				
S: Gaussian	0.06	0.45	0.0086	5.19	75.91%
weights	1	1	1	1	1

**Fig 7:**
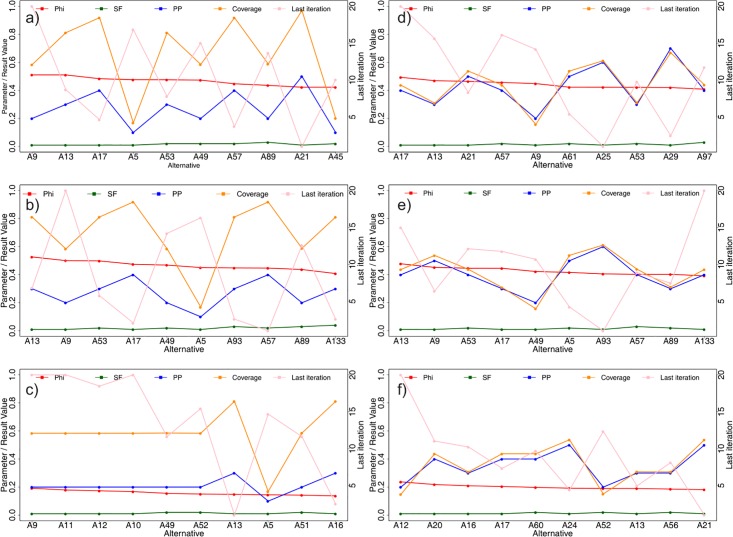
Visual representation of the 10 best strategies in PROMETHEE II rankings for the synthetic (a-c) and real (d-f) networks, based on V-shape preference function with no indifference threshold (a,d), V-shape preference function with indifference threshold (b,e) and Gaussian preference function (c-f).

The first 10 strategies from the ranking obtained with the PROMETHEE II method are presented in Table 4 and on Fig 7a. It can be observed, that all strategies from the list were based on the fastest (and therefore cheapest) degree measure. The leading alternatives A9 and A13, having a difference between their *ϕ* values equal to only 0.003 are very similar. Both use the smallest possible seeding factor of 0.01, whereas the propagation probability is equal to 0.2 and 0.3 for A9 and A13 respectively. As a result of the strategy A9, the campaign took averagely 10.4 iterations and covered 58.37% of the network, whereas for the strategy A13, the campaign was more dynamic (averagely 7.9 iterations) and covered more network (averagely 81.13%). The A17 strategy, ranked third, also uses the seeding factor of 0.01, but the propagation probability was increased to 0.4. It can be observed, that while the process averagely took 7 iterations, the coverage increased intensely to the level of 91.83%. The A5 strategy, ranked 4, uses the computationally cheapest parameters—seeding factor and average propagation probability set to 0.01 and the degree measure used. While the propagation process averagely lasted long, i.e. 9.7 iterations, the obtained coverage was merely 16.89% on average. It can be observed, that the alternatives A17 and A57, ranked 3 and 7 respectively, obtained the same coverage, with equal propagation probability and very similar dynamics of the process. However, in case of A17 the seeding fraction was equal to 0.01 and in case of A57 it was twice as much, i.e. 0.02, for which fact the latter was penalized in the overall ranking. The observation of Fig 7a allows to observe that for the best 10 strategies, the coverage grew along with the propagation probability, but that caused shortening of the process due to its high dynamics.

**Table 4:** Results of the PROMETHEE II method analysis on the synthetic network: a) V-shape preference, b) V-shape preference with indifference threshold, c) Gaussian preference.

Action	*ϕ*	*ϕ*^+^	*ϕ*^−^	Rank	SF	PP	Measure	Last Iter.	Coverage
**a)**	**V-shape, q = 0**								
A9	0.5106	0.6656	0.155	1	0.01	0.2	degree	10.4	58.37%
A13	0.5103	0.6205	0.1102	2	0.01	0.3	degree	7.9	81.13%
A17	0.4833	0.5832	0.0999	3	0.01	0.4	degree	7	91.83%
A5	0.4762	0.6489	0.1727	4	0.01	0.1	degree	9.7	16.89%
A53	0.4758	0.5943	0.1185	5	0.02	0.3	degree	7.7	81.13%
A49	0.4736	0.6365	0.1629	6	0.02	0.2	degree	9.3	58.52%
A57	0.447	0.5566	0.1096	7	0.02	0.4	degree	6.8	91.83%
A89	0.4361	0.613	0.1768	8	0.03	0.2	degree	9	58.83%
A21	0.4226	0.5433	0.1207	9	0.01	0.5	degree	6.2	96.95%
A45	0.4225	0.6054	0.183	10	0.02	0.1	degree	8.2	20.18%
**b)**	**V-shape, q = 50% SD**								
A13	0.5266	0.5686	0.042	1	0.01	0.3	degree	7.9	81.13%
A9	0.5005	0.6349	0.1344	2	0.01	0.2	degree	10.4	58.37%
A53	0.4982	0.5415	0.0433	3	0.02	0.3	degree	7.7	81.13%
A17	0.4745	0.5324	0.0579	4	0.01	0.4	degree	7	91.83%
A49	0.4686	0.6029	0.1343	5	0.02	0.2	degree	9.3	58.52%
A5	0.4508	0.6112	0.1604	6	0.01	0.1	degree	9.7	16.89%
A93	0.4482	0.5019	0.0538	7	0.03	0.3	degree	7.1	81.13%
A57	0.4473	0.5072	0.0599	8	0.02	0.4	degree	6.8	91.83%
A89	0.437	0.5782	0.1411	9	0.03	0.2	degree	9	58.83%
A133	0.4081	0.4819	0.0738	10	0.04	0.3	degree	7.1	81.13%
**c)**	**Gaussian**								
A9	0.1919	0.2103	0.0184	1	0.01	0.2	degree	10.4	58.37%
A11	0.1792	0.1996	0.0204	2	0.01	0.2	closeness	10.4	58.37%
A12	0.1734	0.1948	0.0214	3	0.01	0.2	ev	10.2	58.37%
A10	0.1679	0.1975	0.0296	4	0.01	0.2	betweenness	10.4	58.37%
A49	0.1554	0.174	0.0186	5	0.02	0.2	degree	9.3	58.52%
A52	0.1503	0.1721	0.0217	6	0.02	0.2	ev	9.8	58.37%
A13	0.1486	0.1581	0.0096	7	0.01	0.3	degree	7.9	81.13%
A5	0.1447	0.2064	0.0617	8	0.01	0.1	degree	9.7	16.89%
A51	0.1426	0.1633	0.0207	9	0.02	0.2	closeness	9.3	58.52%
A16	0.1372	0.1497	0.0125	10	0.01	0.3	ev	8.1	81.13%

### 3.4 GAIA analysis

**Fig 8:**
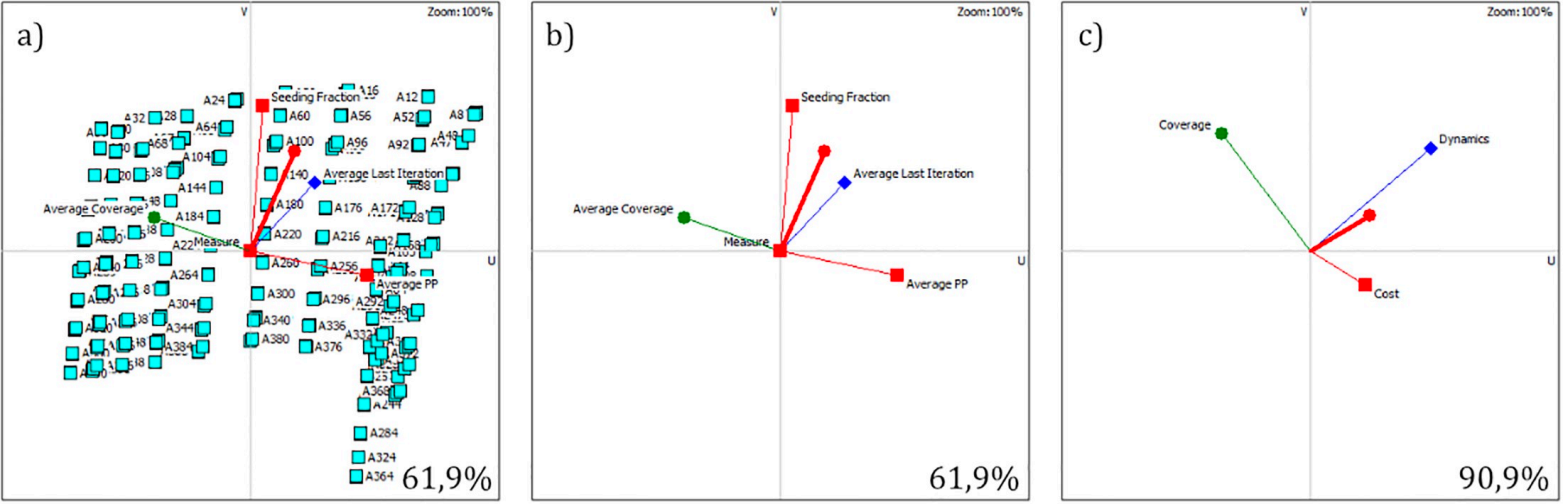
Synthetic network’s GAIA analysis of individual criteria with visible (a) and hidden (b) strategies. GAIA analysis of grouped criteria (c).

The basic PROMETHEE II analysis was followed by the GAIA analysis, which allows to study the relations between criteria, as well as shows which criteria support which strategies. A set of GAIA planes for the PROMETHEE decision problem specified in subsection 3.3 is presented on Fig 8. Fig 8a represents the decision problem with individual criteria and all strategies visible, whereas on Fig 8b the strategies were hidden for better visibility of the criteria vectors. A *δ* = 61.9% quality of the projection was obtained for this GAIA plane. The analysis of Fig 8a allows to observe a very unnatural distribution of the points on the chart. As a matter of fact, this synthetic arrangement of points results from the strategies’ simulative origin and creates a comprehensive grid of possible evaluations of the strategies.

It can be noticed from Fig 8 that the lengths of all criteria vectors is similar, which confirms their similar importance in the evaluation. If one of the vectors was significantly longer, it would mean that the related criterion is more discriminating. The layout of the vectors’ directions allow to note that the cost criterion of the average propagation probability (Par2) is in strong conflict with the average coverage criterion (Eff5) in terms of preference. This confirms an intuitive thesis that the preference for reducing the cost of motivating the members of the social network for passing the seeded information further is in conflict with the preference for maximizing the achieved network coverage. On the other hand, all the cost criteria Par1-Par3 are perpendicular to each other, which means they are generally not related in terms of preference. Last, but not least, the vector representing criterion Eff4 (average last infection iteration) is slightly angled in the direction of both the the vectors representing criterion Par1 (seeding fraction cost) and Par2 (average propagation probability). This means, that the preference for increasing the duration of the campaign may result in selecting similar strategies to the ones in case of the preference for minimizing the costs related to the seeding fraction or the average propagation probability.

One of the advantages of the PROMETHEE methods is their ability to aggregate data into groups and clusters. Fig 8c provides the GAIA analysis for a scenario where the three cost criteria Par1-Par3 were aggregated into a single Cost group, Eff4 into Dynamics group and Eff5 into Coverage group. The *δ* for Fig 8c is very high (90.9%), proving this projection to be very reliable. The analysis of this figure allows to confirm the strong conflict between the Coverage maximization group preference with the Cost minimization preference, however, there is no clear relation between the preferences for the Dynamics and Coverage maximization groups.

### 3.5 Sensitivity analysis

In the scenario analyzed in subsection 3.3, the weights of all criteria were equally set to 1. However, with such a dense grid of alternatives as presented on Fig 8a, it is easy to anticipate that if these preference weights were to change, the ranking of the best strategies would change. For this very reason, MCDA methods provide a tool called sensitivity analysis, which allows to verify the stability of the ranking and to learn how the positions of the alternatives would change if the change in preferences would occur. The results of the performed sensitivity analysis are presented in Tables 5 and 6 for grouped and individual criteria respectively.

**Table 5:** Stability intervals for criteria groups in the PROMETHEE II ranking with V-shape preference function with no indifference threshold for the synthetic network.

Group	Min Weight	Max Weight	Interval
Cost	59.82%	75.15%	15.33%
Dynamics	19.90%	100.00%	80.10%
Coverage	10.14%	20.06%	9.92%

**Table 6:** Stability intervals for individual criteria in the PROMETHEE II ranking with V-shape preference function with no indifference threshold for the synthetic network.

Ranks	1			2			3		
Criterion	Min	Max	Interval	Min	Max	Interval	Min	Max	Interval
Par1	0.00%	100.00%	100.00%	0.00%	100.00%	100.00%	15.24%	100.00%	84.76%
Par2	19.88%	33.51%	13.63%	19.88%	26.70%	6.82%	19.88%	20.99%	1.11%
Par3	1.08%	100.00%	98.92%	1.12%	100.00%	98.88%	4.99%	100.00%	95.01%
Eff4	19.90%	100.00%	80.10%	19.90%	31.17%	11.27%	19.90%	21.39%	1.49%
Eff5	10.14%	20.06%	9.92%	16.21%	20.06%	3.85%	19.43%	20.06%	0.63%

In case of the grouped criteria (Table 5), the initial weights were 60% for the Cost group and 20% each for the Dynamics and Coverage groups. A very wide stability interval, equal to 80.10% can be observed for the Dynamics group, which means that the weight of this criterion can be largely increased and the leading alternative would not change its position. On the other hand, if the weight of the Dynamics group dropped by as little as 0.10%, a change in the ranking leader would occur. Much narrower stability interval is observed for the Cost and Coverage criteria groups, equal to 15.33% and 9.92% respectively. There is possibility to increase the Cost’s weight or decrease the Coverage’s weight slightly without the change of the ranking leader.

If the individual criteria Par1-Eff5 are taken into consideration, wide stability intervals can be observed for the first rank, and, therefore, the ranking stability was also performed for ranks 2 and 3. No changes in the weight of Par1 can cause a change in the rank of the first and second alternative. Only if the Par1 weight drops to below 15.24%, the strategy on rank 3 would be replaced. Similarly, the stability interval for the first three ranks of the Par3 criterion is equal to 95.01%. In case of the Eff4 and Eff5 criteria, the stability intervals for the first rank are the same as in Table 5. For the two leading ranks, the stability interval drops from 80.10% and 9.92% to 11.27% and 3.85% for Eff4 and Eff5 respectively. To sum up, the sensitivity analysis allows to notice that the criteria Par2, Eff4 and Eff5 are most discriminating to the final rankings of the viral advertising strategies for the selected synthetic network.

### 3.6 Uncertainty analysis

The strategies’ evaluations in subsections 3.3 and 3.5 were based on certain data, i.e. on situations that the analyst was always able to specify their preference of one strategy over another regarding to individual criteria. However, such differentiation might not always be possible, especially if the difference between the criteria evaluation values are negligibly small. Therefore, in the subsequent section of the analysis, an uncertainty analysis was performed with the use of the PROMETHEE II method. The evaluation model from subsection 3.3 was modified to use the V-shape preference function with indifference area. Therefore, apart from the *p* threshold, which remained unchanged, the values of the *q* indifference threshold were set to 50% of the standard deviation value for each criterion Par1-Eff5.

The results of the PROMETHEE II analysis with uncertainty taken into account are presented in Table 4b. The analysis of the results allow to observe that strategy A13 overranked the previously leading strategy A9, whereas strategy A17, previously ranked 3, obtained position 4, while position 3 was taken by the strategy 57, previously ranked 5. Strategy A49 advanced from rank 6 to rank 5, whereas the rank of strategy A5 was reduced from 4 to 6. The rank of the strategies A57 and A89 was reduced by one, i.e. from 7 and 8 to 8 and 9 respectively. Two strategies A93 and A133, previously outside the set of the top ten strategies, advanced to ranks 7 and 10 respectively, when uncertainty was taken into consideration. The 10 leading strategies are presented on the chart on Fig 7b.

### 3.7 Gaussian preference function

In the final step of the PROMETHEE II analysis, the sharp V-shaped preference function was replaced with a much softer Gaussian preference function, with its *s* parameter equal to the mean value of each criterion Par1-Eff5. The obtained ranking varies substantially from the ones obtained in subsections 3.3 and 3.6. The first visible difference is that only four of the strategies from the original ranking remained in the top ten positions of the new ranking: A9, A13, A5 and A49 on positions 1, 7, 8 and 5 respectively (see Table 4c and Fig 7c). The remaining ranks were were distributed between strategies based on different centrality measures: closeness (A11, A51), eigenvector centrality (A12, A52, A16) and betweenness (A10). The sensitivity analysis of the newly obtained ranking is presented in Table 7. The analysis of the table allows to observe that Par1 is the least and Eff5 is the most discriminating criterion to the final ranking.

**Table 7:** Stability intervals for individual criteria in the PROMETHEE II ranking with Gaussian preference function.

Ranks	1			2			3		
Criterion	Min	Max	Interval	Min	Max	Interval	Min	Max	Interval
Par1	0.00%	100.00%	100.00%	0.00%	100.00%	100.00%	0.00%	100.00%	100.00%
Par2	0.00%	47.86%	47.86%	0.00%	42.45%	42.45%	0.00%	39.60%	39.60%
Par3	0.00%	100.00%	100.00%	0.00%	41.76%	41.76%	9.55%	35.88%	26.33%
Eff4	1.33%	100.00%	98.67%	7.66%	100.00%	92.34%	9.23%	37.17%	27.94%
Eff5	1.82%	43.84%	42.02%	7.49%	38.45%	30.96%	9.86%	35.64%	25.78%

## 4 Evaluation of viral marketing campaign strategies in a real network

### 4.1 Overview of the simulations in the real network

In the second stage of the empirical research, the proposed framework was used to evaluate the viral marketing campaign strategies within a real network [77]. Similarly to the study in subsection 3.2, ten simulation scenarios were generated for the network, in order to ascertain the repeatability of the results regardless of the simulated parameters, as well as 400 various sets of parameters for the criteria Par1-Par3 as in subsection 3.2 were tested in the simulations. In case of the real network, the values of the Par3 criterion were as follows: degree [0.0200], betweenness [3.6900], closeness [2.1200], eigenvector centrality [0.030]. The output of the simulations was used to obtain the average performance values for Eff4 and Eff5 criteria for each of the 400 sets of parameters.

### 4.2 PROMETHEE II analysis

The results of simulations from subsection 4.1 were used to build the performance table for the PROMETHEE II analysis. As in section 3.3, V-shape preference function was used for modeling the comparison preferences, with the indifference threshold *q* = 0 and the preference threshold *p* equal to the standard deviation value for each criterion Par1-Eff5. The direction of the preference functions were as in subsection 3.3, to allow comparison of the results on the synthetic network and the real network (see Table 8a). The ten strategies which ranked highest are presented in Table 9a and Fig 7d.

**Table 8:** PROMETHEE II parameters for the real network.

	Par1	Par2	Par3	Eff4	Eff5
	**Statistics**				
Minimum	0.01	0.01	0.02	1	1.00%
Maximum	0.1	0.9	3.69	21	74.79%
Average	0.06	0.45	1.4650	9.48	44.53%
Standard Dev.	0.03	0.29	1.5433	3.80	24.14%
**a)**	**V-shape, q = 0**				
Q: indifference	0	0	0	0	0
P: preference	0.03	0.29	1.5433	3.80	24.14%
weights	1	1	1	1	1
**b)**	**V-shape, q = 50% SD**				
Q: indifference	0.015	0.145	0.0009	1.90	12.07%
P: preference	0.03	0.29	0.0018	3.80	24.14%
weights	1	1	1	1	1
**c)**	**Gaussian**				
S: Gaussian	0.06	0.45	1.4650	9.48	44.53%
weights	1	1	1	1	1

**Table 9:** Results of the PROMETHEE II method analysis on the real network: a) V-shape preference, b) V-shape preference with indifference threshold, c) Gaussian preference.

Action	*ϕ*	*ϕ*^+^	*ϕ*^−^	Rank	SF	PP	Measure	Last Iter.	Coverage
**a)**	**V-shape, q = 0**								
A17	0.493	0.6396	0.1466	1	0.01	0.4	degree	16.2	43.74%
A13	0.4694	0.6273	0.1579	2	0.01	0.3	degree	15.3	30.92%
A21	0.4645	0.6033	0.1387	3	0.01	0.5	degree	13.8	53.73%
A57	0.4567	0.6109	0.1542	4	0.02	0.4	degree	15.4	43.84%
A9	0.4483	0.6123	0.1641	5	0.01	0.2	degree	15	15.75%
A61	0.4227	0.5715	0.1488	6	0.02	0.5	degree	13.2	53.82%
A25	0.4224	0.5671	0.1446	7	0.01	0.6	degree	12.3	61.16%
A53	0.4218	0.5897	0.1679	8	0.02	0.3	degree	14.1	31.21%
A29	0.4202	0.5678	0.1476	9	0.01	0.7	degree	12.6	66.82%
A97	0.4085	0.5783	0.1699	10	0.03	0.4	degree	14.5	43.94%
**b)**	**V-shape, q = 50% SD**								
A17	0.478	0.5942	0.1162	1	0.01	0.4	degree	16.2	43.74%
A21	0.452	0.5481	0.0961	2	0.01	0.5	degree	13.8	53.73%
A57	0.4463	0.5625	0.1162	3	0.02	0.4	degree	15.4	43.84%
A13	0.446	0.5774	0.1314	4	0.01	0.3	degree	15.3	30.92%
A9	0.4227	0.5607	0.138	5	0.01	0.2	degree	15	15.75%
A61	0.4164	0.5142	0.0978	6	0.02	0.5	degree	13.2	53.82%
A25	0.4057	0.5055	0.0999	7	0.01	0.6	degree	12.3	61.16%
A97	0.403	0.5267	0.1237	8	0.03	0.4	degree	14.5	43.94%
A53	0.4029	0.5355	0.1325	9	0.02	0.3	degree	14.1	31.21%
A20	0.3922	0.5585	0.1663	10	0.01	0.4	ev	17.6	43.73%
**c)**	**Gaussian**								
A12	0.2372	0.2934	0.0562	1	0.01	0.2	ev	21	14.87%
A20	0.2182	0.2432	0.025	2	0.01	0.4	ev	17.6	43.73%
A16	0.2092	0.2433	0.0341	3	0.01	0.3	ev	17.3	30.78%
A17	0.2041	0.2291	0.025	4	0.01	0.4	degree	16.2	43.74%
A60	0.1978	0.2231	0.0253	5	0.02	0.4	ev	17.1	43.74%
A24	0.1922	0.2188	0.0267	6	0.01	0.5	ev	15.1	53.73%
A52	0.1902	0.2464	0.0562	7	0.02	0.2	ev	18.1	15.08%
A13	0.19	0.2241	0.0341	8	0.01	0.3	degree	15.3	30.92%
A56	0.1859	0.2204	0.0345	9	0.02	0.3	ev	16.5	30.81%
A21	0.1816	0.2086	0.027	10	0.01	0.5	degree	13.8	53.73%

The analysis of Table 9a allows to observe that similarly to the strategies obtained on the synthetic network, all 10 best strategies are based on the cheapest ranking measure, i.e. degree. The leading strategy A17 is based on a low seeding fraction (0.1), and mediocre propagation probability (0.4), which leads to very long process (16.2 iterations), but mediocre coverage (43.74%). The strategies A13 and A21 obtained a very close *ϕ* values, 0.4694 and 0.4645 respectively. The analysis of 7 shows that whilst having the same seeding fraction (0.1), they differ in the propagation probability and obtained duration and coverage. The higher-ranked strategy A13 uses lower propagation probability (0.3 compared to 0.5) and lasts longer (averagely 15.3 iterations compared to 13.8 iterations), but results in much lower coverage (30.92% compared to 53.73%). A further analysis of Fig 7d allows to observe that for the 10 leading strategies, increasing the propagation probability results in the increase of the coverage and in the reduction of the count of the propagation process iterations.

**Fig 9:**
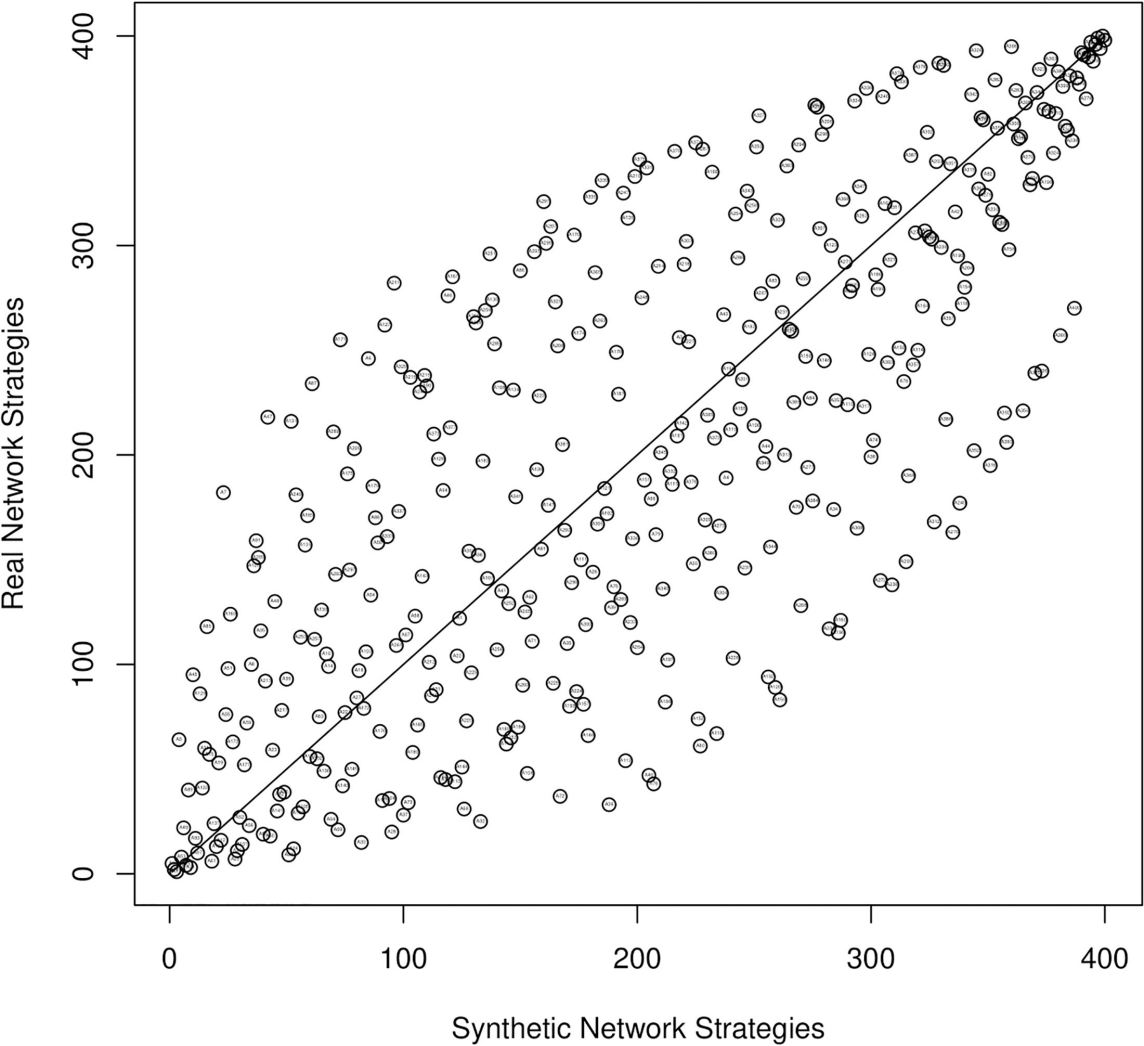
Comparison of the ranks of strategies obtained based on a synthetic and a real network [77].

It can be observed that six of the strategies, i.e. A17, A13, A21, A57, A9 and A53 also occurred on the top 10 positions of the ranking obtained from the synthetic network in subsection 3.3. A further comparison of the results allows to note that the both rankings of strategies are highly correlated, with the Pearson correlation coefficient equal to 0.7589. The high correlation can be visually confirmed on the chart on Fig 9. The chart shows the positions of each strategy obtained in the ranking based on the synthetic network (x axis) and on the real network (y axis). The closer the strategy is plotted to the diagonal line on the chart, the more similar was the rank of the strategy in each ranking. The analysis of the figure clearly shows a similarity of the evaluations of strategies in both rankings.

### 4.3 GAIA analysis

**Fig 10:**
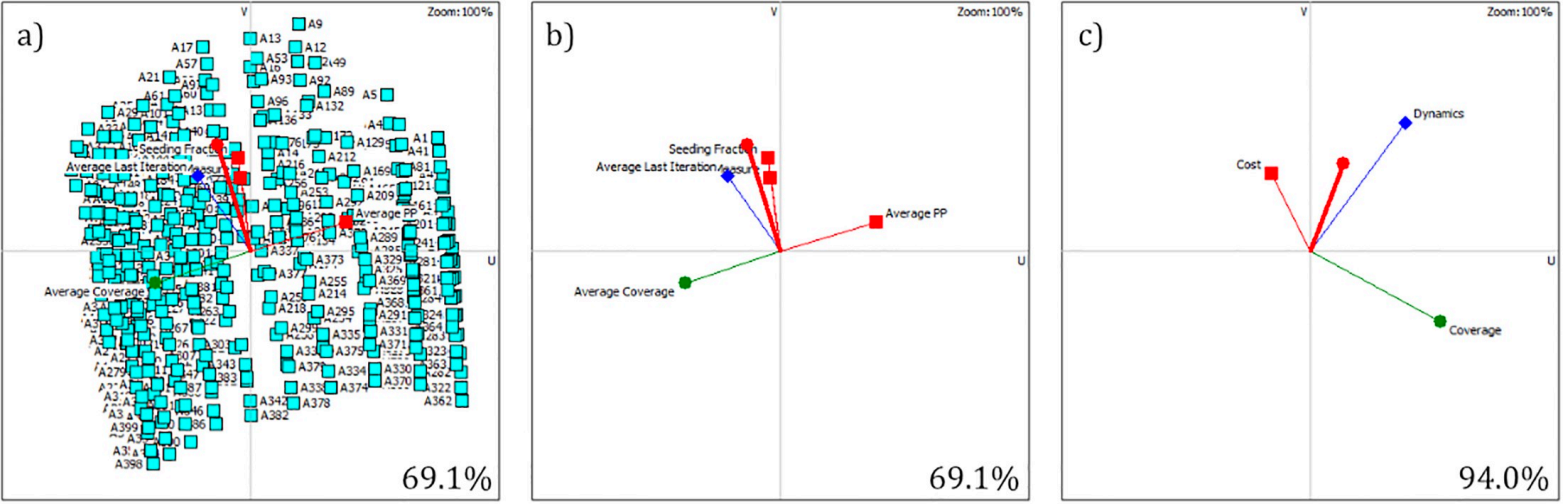
Real network’s GAIA analysis of individual criteria with visible (a) and hidden (b) strategies. GAIA analysis of grouped criteria (c).

The basic PROMETHEE II analysis was followed by the GAIA analysis. A set of GAIA planes for the PROMETHEE decision problem specified in subsection 4.2 is presented on Fig 8. Fig 10a represents the decision problem with individual criteria and all strategies visible, whereas on Fig 10b the strategies were hidden for better clarity of the criteria vectors’ analysis. A *δ* = 69.1% quality of the projection was obtained for this GAIA plane. The analysis of Fig 10a allows to observe that although the grid structure of the strategies similar to the one from subsection 3.4 is still noticeable, much more randomness can be observed, especially in the II and III quadrants, i.e. where the Eff4 and Eff5 preference vectors point to. This higher randomness level in the grid results from the fact that here the values of the the Eff4 and Eff5 criteria are taken from the empirical measurement based on a real network, as opposed to the synthetic network in subsection 3.4.

The analysis of Fig 10b shows that again the preference for the Par2 criterion (average propagation probability) is in strong conflict with the preference for the Eff5 criterion (average coverage). On the other hand, the vectors for criteria Par1, Par3 and Eff4 are pointing similar directions which indicates similarity in preference of these three criteria. In contrast to what was observed in subsection 3.4, the vectors of Par1 and Par3 criteria (seeding fraction and ranking generation measure) are no longer perpendicular to each other. Instead, they point the same direction, thus demonstrating a similarity in preference of reducing the cost of both these criteria. This can be caused by a considerably higher value of the standard deviation for the values of the criterion Par3 for the real network compared to the synthetic network. Moreover, it is significant to keep in mind that the action of generating the rankings of network nodes before seeding is strongly related to the action of seeding limited fraction of the best nodes from such obtained ranking.

When the criteria Par1-Eff5 are aggregated into three groups again, i.e. Cost, Dynamics and Coverage (see Fig 10c with *δ* = 94.0%), a very similar relation of the groups to the one obtained in subsection 3.4 can be observed (compare with Fig 8c). There is a strong conflict between the Cost minimization and Coverage maximization criteria, but the Coverage and Dynamics maximization criteria are not highly related in terms of preference. This time, however, a very minute similarity between the preference for the Dynamics maximization and Cost minimization criteria can be observed.

### 4.4 Sensitivity analysis

Similar to subsection 3.5, a sensitivity analysis was performed also for the real network. The results of the performed analysis are presented in Tables 10 and 11 for grouped and individual criteria respectively.

**Table 10:** Stability intervals for criteria groups in the PROMETHEE II ranking with V-shape preference function with no indifference threshold for the real network.

Group	Min Weight	Max Weight	Interval
Cost	37.17%	69.97%	32.80%
Dynamics	7.00%	62.99%	55.99%
Coverage	12.86%	27.85%	14.99%

**Table 11:** Stability intervals for individual criteria in the PROMETHEE II ranking with V-shape preference function with no indifference threshold for the real network.

Ranks	1			2			3		
Criterion	Min	Max	Interval	Min	Max	Interval	Min	Max	Interval
Par1	0.00%	100.00%	100.00%	11.58%	100.00%	88.42%	15.03%	100.00%	84.97%
Par2	8.98%	27.97%	18.99%	19.01%	27.52%	8.51%	19.01%	22.09%	3.08%
Par3	2.69%	100.00%	97.31%	7.96%	100.00%	92.04%	8.99%	100.00%	91.01%
Eff4	7.00%	62.99%	55.99%	17.22%	49.18%	31.96%	17.22%	23.88%	6.66%
Eff5	12.86%	27.85%	14.99%	13.60%	20.74%	7.14%	18.36%	20.74%	2.38%

In case of the grouped criteria (Table 10), the initial weights were 60% for the Cost group and 20% each for the Dynamics and Coverage groups. The widest stability interval, equal to 55.99% can be observed for the Dynamics group, which means that the weight of this criterion can be decreased by 13% or increased by 42.99% and the leading alternative would not change its position. A narrower stability interval can be observed for the Cost criteria group, equal to 32.80%. The weight of this group can be increased by 9.97% or decreased by 22.83% without the change of the leader strategy. For the Coverage criteria groups, the stability interval is equal to 14.99% and its weight can be reduced to 12.86% or increased to 27.85% without a change of the leader strategy. When compared to the results obtained for the strategies based on the synthetic network (see Table 5), a 0.9292 correlation coefficient is obtained for the intervals. However, the Dynamics cluster interval is narrower, yet the Cost and Coverage cluster intervals are wider.

If the individual criteria Par1-Eff5 are taken into consideration, wide stability intervals can be observed for the first rank, and, therefore, the ranking stability was also performed for ranks 2 and 3. No changes in the weight of Par1 can cause a change in the rank of the first strategy, however, if the Par1 weight drops to below 11.58%, the strategy on rank 2 would be replaced. Similarly, the stability interval for the first three ranks of the Par3 criterion is equal to 91.01%. In case of the Eff4 and Eff5 criteria, the stability intervals for the first rank are the same as in Table 10. If two leading ranks were considered instead of a single leading rank, the stability interval would drop from 55.99% and 14.99% to 49.18% and 20.74% for Eff4 and Eff5 respectively. Again, as in subsection 3.5, the sensitivity analysis allows to notice that the criteria Par2, Eff4 and Eff5 are most discriminating to the final rankings of the viral advertising strategies. When the results for the real network are compared with the results for the synthetic network, correlation indexes of 0.9217, 0.4928 and 0.3546 are obtained respectively for the stability intervals for up to 1, up to 2 and up to 3 leading strategies.

### 4.5 Uncertainty analysis

An uncertainty analysis similar to the one in subsection 3.6 was also performed for the real network. Again, the *q* indifference threshold for all criteria was set to 50% of their standard deviation values (see Table 8b). The results of the uncertainty analysis are presented in Table 9b.

The analysis of the results allow to observe that even when the uncertainty is taken into account, strategy A17 remains the leading one for the real network. The order of the subsequent strategies changes from A13, A21 and A57 to A21, A56 and A13 on ranks 2 to 4. Strategies A9, A61 and A25 remained unchanged on positions 5 to 7. Strategy A53 was degraded from rank 8 to rank 9, whereas the rank 8 was given to the previously 10th strategy A97. When the uncertainty was taken into consideration, strategy A20, previously outside of the set of the top ten strategies, obtained rank 10. The ten leading strategies are presented on the chart on Fig 7e.

### 4.6 Gaussian preference function

In the final step of the analysis, the sharp V-shaped preference function was replaced with the Gaussian preference function, with its *s* parameter equal to the mean value of each criterion Par1-Eff5. In contrast to the rankings from subsections 4.2 and 4.5, the obtained ranking contains mostly strategies based on the eigenvector centrality measure. The only three strategies based on the degree measure are A17, A13 and A21 (ranks 4, 8 and 10 respectively). The analysis of Fig 7f shows that the highest-appraised strategy A12 is based on a small value of seeding fraction (0.01 for Par1) and long duration (21 iterations in Eff4), however the obtained coverage is very small (14.87% for Eff5).

The results of the sensitivity analysis for the ranking is presented in Table 12. It can be observed, that the Par3 criterion is the least discriminating one when the Gaussian preference function is used and its weight can be vastly modified (up to 86.12%) without considerable changes in ranking. However, if the weight of the Eff5 criterion grew by 5.17%, the leading strategy in the ranking would change.

**Table 12:** Stability intervals for individual criteria in the PROMETHEE II ranking with Gaussian preference function for the real network.

Ranks	1			2			3		
Criterion	Min	Max	Interval	Min	Max	Interval	Min	Max	Interval
Par1	0.00%	100.00%	100.00%	0.00%	100.00%	100.00%	5.85%	100.00%	94.15%
Par2	10.63%	59.54%	48.91%	10.63%	27.19%	16.56%	15.23%	27.19%	11.96%
Par3	0.00%	97.48%	97.48%	0.00%	94.34%	94.34%	0.00%	86.12%	86.12%
Eff4	11.05%	100.00%	98.95%	11.05%	60.75%	49.70%	11.92%	44.57%	32.65%
Eff5	0.00%	25.17%	25.17%	13.53%	25.17%	11.64%	13.53%	23.27%	9.74%

## 5 Conclusions

Recently, marketers have put more and more efforts to perceive the positive user experience within online platforms. While intrusive marketing techniques resulted in increased interest in software focused on blocking advertising content marketers, a new demand to focus on searching for more sustainable solutions appeared. The overall number of contacted customers can be less important than the real interest in products and a proper specification of the campaign intensity. In the area of viral marketing and information spreading processes, the highest attention was put on increasing campaign coverage with the use of seeding methods and techniques increasing the propagation probability like incentives and other ways to motivate customers to spread the content.

The approach presented in this paper shows a framework based on multi-criteria decision support, targeted on planning and evaluation of marketing campaigns with different preferences and criteria taken into account. The results showed how multi-criteria evaluation of results can affect strategies, campaign parameters and allocated budgets. The presented approach makes it possible to perform an evaluation of different scenarios within simulated environment before the campaign within a real environment begins. The empirical study showed that the characteristics of information spreading processes within the network sample selected according to network measures’ distributions are similar to those observed within a real network. Various scenarios can be tested without interaction with real environments.

During the empirical study, an example viral marketing campaign was planned for an actual real network. Based on the real network parameters, a corresponding synthetic model was selected. Preference modeling and a profound multi-objective decision analysis were performed, which resulted in the selection of the best strategy in the context of the previously modeled preferences.

The research has identified possible areas of improvement and future works. First of all, the decision support system utilized in the presented framework was based on a set of five criteria. This set can be expanded to provide more precise evaluations. Secondly, future works include a more detailed evaluation of the relations between the processes within real networks and theoretical structures generated with different parameters. Another direction can be a sampling of real networks and performing simulations on samples of real networks instead of theoretical network models.

## Supporting information

S1 File Microsoft Excel spreadsheet containing partial results used during the research. (XLSX)

## References

[1] Hanna R, Rohm A, Crittenden VL. WeÔÇÖre all connected: The power of the social media ecosystem. Business horizons. 2011;54(3):265–273. 10.1016/j.bushor.2011.01.007

[2] Watts DJ, Peretti J, Frumin M. Viral marketing for the real world. Harvard Business School Pub.; 2007.

[3] Iribarren JL, Moro E. Impact of human activity patterns on the dynamics of information diffusion. Physical review letters. 2009;103(3):038702 10.1103/PhysRevLett.103.038702 19659326

[4] Berger J, Milkman KL. What makes online content viral? Journal of marketing research. 2012;49(2):192–205. 10.1509/jmr.10.0353

[5] Ho JY, Dempsey M. Viral marketing: Motivations to forward online content. Journal of Business research. 2010;63(9-10):1000–1006. 10.1016/j.jbusres.2008.08.010

[6] Zhang X, Han DD, Yang R, Zhang Z. UsersÔÇÖ participation and social influence during information spreading on Twitter. PloS one. 2017;12(9):e0183290 10.1371/journal.pone.0183290 28902906 PMC5597198

[7] Hinz O, Skiera B, Barrot C, Becker JU. Seeding strategies for viral marketing: An empirical comparison. Journal of Marketing. 2011;75(6):55–71. 10.1509/jm.10.0088

[8] Liu-Thompkins Y. Seeding viral content: The role of message and network factors. Journal of Advertising Research. 2012;52(4):465–478. 10.2501/JAR-52-4-465-478

[9] Kandhway K, Kuri J. How to run a campaign: Optimal control of SIS and SIR information epidemics. Applied Mathematics and Computation. 2014;231:79–92. 10.1016/j.amc.2013.12.164

[10] Kiss C, Bichler M. Identification of influencers: measuring influence in customer networks. Decision Support Systems. 2008;46(1):233–253. 10.1016/j.dss.2008.06.007

[11] Nejad MG, Amini M, Babakus E. Success factors in product seeding: The role of homophily. Journal of Retailing. 2015;91(1):68–88. 10.1016/j.jretai.2014.11.002

[12] Bampo M, Ewing MT, Mather DR, Stewart D, Wallace M. The effects of the social structure of digital networks on viral marketing performance. Information systems research. 2008;19(3):273–290. 10.1287/isre.1070.0152

[13] Stieglitz S, Dang-Xuan L. Emotions and information diffusion in social media: sentiment of microblogs and sharing behavior. Journal of management information systems. 2013;29(4):217–248. 10.2753/MIS0742-1222290408

[14] Dobele A, Lindgreen A, Beverland M, Vanhamme J, Van Wijk R. Why pass on viral messages? Because they connect emotionally. Business Horizons. 2007;50(4):291–304. 10.1016/j.bushor.2007.01.004

[15] Camarero C, San José R. Social and attitudinal determinants of viral marketing dynamics. Computers in Human Behavior. 2011;27(6):2292–2300. 10.1016/j.chb.2011.07.008

[16] Salehi M, Sharma R, Marzolla M, Magnani M, Siyari P, Montesi D. Spreading processes in multilayer networks. IEEE Transactions on Network Science and Engineering. 2015;2(2):65–83. 10.1109/TNSE.2015.2425961

[17] Michalski R, Kajdanowicz T, Bródka P, Kazienko P. Seed selection for spread of influence in social networks: Temporal vs. static approach. New Generation Computing. 2014;32(3-4):213–235. 10.1007/s00354-014-0402-9

[18] Helm S. Viral marketing-establishing customer relationships by’word-of-mouse’. Electronic markets. 2000;10(3):158–161.

[19] Cruz D, Fill C. Evaluating viral marketing: isolating the key criteria. Marketing Intelligence & Planning. 2008;26(7):743–758. 10.1108/02634500810916690

[20] Welker CB. The paradigm of viral communication. Information Services & Use. 2002;22(1):3–8. 10.3233/ISU-2002-22102

[21] Ni Y, Shi Q, Wei Z. Optimizing influence diffusion in a social network with fuzzy costs for targeting nodes. Journal of Ambient Intelligence and Humanized Computing. 2017;8(5):819–826. 10.1007/s12652-017-0552-y

[22] Rogers EM. Diffusion of innovations. Simon and Schuster; 2010.

[23] Pfitzner R, Garas A, Schweitzer F. Emotional Divergence Influences Information Spreading in Twitter. ICWSM. 2012;12:2–5.

[24] Chen W, Wang C, Wang Y. Scalable influence maximization for prevalent viral marketing in large-scale social networks. In: Proceedings of the 16th ACM SIGKDD international conference on Knowledge discovery and data mining. ACM; 2010. p. 1029–1038.

[25] Wasserman S, Faust K. Social network analysis: Methods and applications. vol. 8 Cambridge university press; 1994.

[26] Kempe D, Kleinberg J, Tardos É. Maximizing the spread of influence through a social network. In: Proceedings of the ninth ACM SIGKDD international conference on Knowledge discovery and data mining. ACM; 2003. p. 137–146.

[27] Jankowski J, Bródka P, Kazienko P, Szymanski B, Michalski R, Kajdanowicz T. Balancing Speed and Coverage by Sequential Seeding in Complex Networks. arXiv preprint arXiv:160907526. 2016;. 10.1038/s41598-017-00937-8 PMC5429852 28420880

[28] He JL, Fu Y, Chen DB. A novel top-k strategy for influence maximization in complex networks with community structure. PloS one. 2015;10(12):e0145283 10.1371/journal.pone.0145283 26682706 PMC4689492

[29] Jankowski J. Dynamic rankings for seed selection in complex networks: Balancing costs and coverage. Entropy. 2017;19(4):170 10.3390/e19040170

[30] Zhang JX, Chen DB, Dong Q, Zhao ZD. Identifying a set of influential spreaders in complex networks. Scientific reports. 2016;6:27823 10.1038/srep27823 27296252 PMC4906276

[31] Kitsak M, Gallos LK, Havlin S, Liljeros F, Muchnik L, Stanley HE, et al Identification of influential spreaders in complex networks. Nature physics. 2010;6(11):888 10.1038/nphys1746

[32] Sankar CP, Asharaf S, Kumar KS. Learning from bees: An approach for influence maximization on viral campaigns. PloS one. 2016;11(12):e0168125 10.1371/journal.pone.0168125 27992472 PMC5167354

[33] Seeman L, Singer Y. Adaptive seeding in social networks. In: Foundations of Computer Science (FOCS), 2013 IEEE 54th Annual Symposium on. IEEE; 2013. p. 459–468.

[34] Granell C, Gómez S, Arenas A. Competing spreading processes on multiplex networks: awareness and epidemics. Physical review E. 2014;90(1):012808 10.1103/PhysRevE.90.012808 25122343

[35] Barabási AL, Albert R. Emergence of scaling in random networks. science. 1999;286(5439):509–512. 10.1126/science.286.5439.509 10521342

[36] ERDdS P, R&WI A. On random graphs I. Publ Math Debrecen. 1959;6:290–297.

[37] Watts DJ, Strogatz SH. Collective dynamics of ÔÇśsmall-worldÔÇÖnetworks. nature. 1998;393(6684):440 10.1038/30918 9623998

[38] Massaro E, Bagnoli F. Epidemic spreading and risk perception in multiplex networks: a self-organized percolation method. Physical Review E. 2014;90(5):052817 10.1103/PhysRevE.90.052817 25493844

[39] Wei X, Chen S, Wu X, Feng J, Lu Ja. A unified framework of interplay between two spreading processes in multiplex networks. EPL (Europhysics Letters). 2016;114(2):26006 10.1209/0295-5075/114/26006

[40] Wei X, Wu X, Chen S, Lu Ja, Chen G. Cooperative epidemic spreading on a two-layered interconnected network. SIAM Journal on Applied Dynamical Systems. 2018;17(2):1503–1520. 10.1137/17M1134202

[41] Brans JP, Mareschal B. PROMETHEE methods In: Multiple criteria decision analysis: state of the art surveys. Springer; 2005 p. 163–186.

[42] Roy B, Słowiński R. Questions guiding the choice of a multicriteria decision aiding method. EURO Journal on Decision Processes. 2013;1(1-2):69–97. 10.1007/s40070-013-0004-7

[43] Roy B. Paradigms and challenges In: Multiple criteria decision analysis: state of the art surveys. Springer; 2005 p. 3–24.

[44] Ceballos B, Lamata MT, Pelta DA. A comparative analysis of multi-criteria decision-making methods. Progress in Artificial Intelligence. 2016;5(4):315–322. 10.1007/s13748-016-0093-1

[45] Wątróbski J, Jankowski J, Ziemba P, Karczmarczyk A, Zioło M. Generalised framework for multi-criteria method selection. Omega. 2018;. 10.1016/j.dib.2018.12.015 PMC6327857 30671511

[46] Mardani A, Jusoh A, Zavadskas EK. Fuzzy multiple criteria decision-making techniques and applications–Two decades review from 1994 to 2014. Expert systems with Applications. 2015;42(8):4126–4148. 10.1016/j.eswa.2015.01.003

[47] Celik M, Er ID. Fuzzy axiomatic design extension for managing model selection paradigm in decision science. Expert Systems with Applications. 2009;36(3):6477–6484. 10.1016/j.eswa.2008.07.038

[48] Kurka T, Blackwood D. Selection of MCA methods to support decision making for renewable energy developments. Renewable and Sustainable Energy Reviews. 2013;27:225–233. 10.1016/j.rser.2013.07.001

[49] Wang X, Triantaphyllou E. Ranking irregularities when evaluating alternatives by using some ELECTRE methods. Omega. 2008;36(1):45–63. 10.1016/j.omega.2005.12.003

[50] Peng Y, Wang G, Wang H. User preferences based software defect detection algorithms selection using MCDM. Information Sciences. 2012;191:3–13. 10.1016/j.ins.2010.04.019

[51] Chang YH, Yeh CH, Chang YW. A new method selection approach for fuzzy group multicriteria decision making. Applied Soft Computing. 2013;13(4):2179–2187. 10.1016/j.asoc.2012.12.009

[52] Kolios A, Mytilinou V, Lozano-Minguez E, Salonitis K. A comparative study of multiple-criteria decision-making methods under stochastic inputs. Energies. 2016;9(7):566 10.3390/en9070566

[53] Guitouni A, Martel JM. Tentative guidelines to help choosing an appropriate MCDA method. European Journal of Operational Research. 1998;109(2):501–521. 10.1016/S0377-2217(98)00073-3

[54] Ulengin F, Topcu YI, Sahin SO. An artificial neural network approach to multicriteria model selection In: Multiple Criteria Decision Making in the New Millennium. Springer; 2001 p. 101–110.

[55] Vansnick JC. On the problem of weights in multiple criteria decision making (the noncompensatory approach). European Journal of Operational Research. 1986;24(2):288–294. 10.1016/0377-2217(86)90051-2

[56] Corrente S, Greco S, Słowiński R. Multiple criteria hierarchy process with ELECTRE and PROMETHEE. Omega. 2013;41(5):820–846. 10.1016/j.omega.2012.10.009

[57] Wątróbski J, Ziemba E, Karczmarczyk A, Jankowski J. An Index to Measure the Sustainable Information Society: The Polish Households Case. Sustainability. 2018;10(9).

[58] Roy B, Bouyssou D. Aide multicritère à la décision: méthodes et cas. Economica Paris; 1993.

[59] Kullback S, Leibler RA. On Information and Sufficiency. Ann Math Statist. 1951;22(1):79–86. 10.1214/aoms/1177729694

[60] Wang C, Chen W, Wang Y. Scalable influence maximization for independent cascade model in large-scale social networks. Data Mining and Knowledge Discovery. 2012;25(3):545–576. 10.1007/s10618-012-0262-1

[61] Mochalova A, Nanopoulos A. Non-intrusive Viral Marketing Based on Percolation Centrality. In: ECIS; 2015.

[62] Zhang P, Chen W, Sun X, Wang Y, Zhang J. Minimizing seed set selection with probabilistic coverage guarantee in a social network. In: Proceedings of the 20th ACM SIGKDD international conference on Knowledge discovery and data mining. ACM; 2014. p. 1306–1315.

[63] Dinh TN, Zhang H, Nguyen DT, Thai MT. Cost-effective viral marketing for time-critical campaigns in large-scale social networks. IEEE/ACM Transactions on Networking (ToN). 2014;22(6):2001–2011. 10.1109/TNET.2013.2290714

[64] Abebe R, Adamic L, Kleinberg J. Mitigating overexposure in viral marketing. arXiv preprint arXiv:170904123. 2017;.

[65] Shakarian P, Paulo D. Large social networks can be targeted for viral marketing with small seed sets. In: Proceedings of the 2012 International Conference on Advances in Social Networks Analysis and Mining (ASONAM 2012). IEEE Computer Society; 2012. p. 1–8.

[66] Wu W, Du DZ, et al. Coupon Advertising in Online Social Systems: Algorithms and Sampling Techniques. arXiv preprint arXiv:180206946. 2018;.

[67] Kotnis B, Sunny A, Kuri J. Incentivized Campaigning in Social Networks. IEEE/ACM Transactions on Networking. 2017;25(3):1621–1634. 10.1109/TNET.2016.2645281

[68] Zhang B, Qian Z, Li W, Lu S. Pricing strategies for maximizing viral advertising in social networks. In: International conference on database systems for advanced applications. Springer; 2015. p. 418–434.

[69] Doo M, Liu L. Probabilistic diffusion of social influence with incentives. IEEE Transactions on Services Computing. 2014;7(3):387–400. 10.1109/TSC.2014.2310216

[70] Tang J, Tang X, Yuan J. Profit maximization for viral marketing in online social networks. In: Network Protocols (ICNP), 2016 IEEE 24th International Conference on. IEEE; 2016. p. 1–10.

[71] Long C, Wong RCW. Viral marketing for dedicated customers. Information Systems. 2014;46:1–23. 10.1016/j.is.2014.05.003

[72] Hu H, Wen Y, Feng S. Budget-efficient viral video distribution over online social networks: Mining topic-aware influential users. IEEE Transactions on Circuits and Systems for Video Technology. 2018;28(3):759–771. 10.1109/TCSVT.2016.2620152

[73] Nguyen HT, Dinh TN, Thai MT. Cost-aware targeted viral marketing in billion-scale networks. In: INFOCOM 2016-The 35th Annual IEEE International Conference on Computer Communications, IEEE. IEEE; 2016. p. 1–9.

[74] Yue W, WeiJing H, Lang Z, TengJiao W, DongQing Y. Influence maximization with limit cost in social network. SCIENCE CHINA-INFORMATION SCIENCES. 2013;56(7).

[75] Goyal A, Bonchi F, Lakshmanan LV, Venkatasubramanian S. On minimizing budget and time in influence propagation over social networks. Social network analysis and mining. 2013;3(2):179–192. 10.1007/s13278-012-0062-z

[76] Jankowski J, Szymanski BK, Kazienko P, Michalski R, Bródka P. Probing Limits of Information Spread with Sequential Seeding. Scientific reports. 2018;8(1):13996 10.1038/s41598-018-32081-2 30228338 PMC6143613

[77] Newman ME. The structure of scientific collaboration networks. Proceedings of the national academy of sciences. 2001;98(2):404–409. 10.1073/pnas.98.2.404 PMC14598 11149952

